# Iron‐Catalyzed Intramolecular Arene C(sp^2^)−H Amidations under Mechanochemical Conditions

**DOI:** 10.1002/anie.202204874

**Published:** 2022-06-10

**Authors:** Peng Shi, Yongliang Tu, Deshen Kong, Peng Wu, Ding Ma, Carsten Bolm

**Affiliations:** ^1^ Institute of Organic Chemistry RWTH Aachen University Landoltweg 1 52074 Aachen Germany

**Keywords:** Amidation, Ball Mill, Dihydroquinolinone, Iron Catalysis, Mechanochemistry

## Abstract

In a ball mill, FeBr_3_‐catalyzed intramolecular amidations lead to 3,4‐dihydro‐2(1*H*)‐quinolinones in good to almost quantitative yields. The reactions do not require a solvent and are easy to perform. No additional ligand is needed for the iron catalyst. Both 4‐substituted aryl and β‐substituted dioxazolones provide products with high selectivity. Mechanistically, an electrophilic spirocyclization followed by C−C migration explains the formation of rearranged products.

## Graphical abstract

A mechanochemical approach towards 3,4‐dihydro‐2(1*H*)‐quinolinones by catalysis with FeBr_3_ has been developed. The solvent‐free processes start from readily available dioxazolones, which undergo intramolecular amidation reactions providing the products in good to very high yields. Practically, the reactions are easy to perform, proceed within a short period of time and require only standard ball milling equipment.

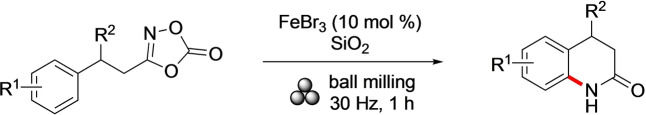

3,4‐Dihydro‐2(1*H*)‐quinolinones are important structural motifs occurring in natural products,^[1]^ biologically active compounds,^[2]^ and drugs.^[3]^ Over the past decades, various synthetic approaches towards 3,4‐dihydro‐2(1*H*)‐quinolinones have been developed, including Friedel–Crafts alkylations,^[4]^ transition metal catalyses,^[5]^ and radical reactions.^[6]^ Although these methods can be efficient in given synthetic settings, they commonly require prefunctionalized substrates resulting from multi‐step syntheses or involve complex reaction conditions, which limits their applicability. Hence, the development of easy‐to‐perform syntheses of 3,4‐dihydro‐2(1*H*)‐quinolinones has remained an attractive goal.

In 2018, Chang and co‐workers reported iridium‐catalyzed intramolecular arene C(sp^2^)‐H amidation reactions affording 3,4‐dihydro‐2(1*H*)‐quinolinones with dioxazolones as convenient acyl nitrene precursors (Scheme 1, top).[^7^, ^8^, ^9^, ^10^] An analogous ruthenium catalysis was reported by Yu and co‐workers in 2021.^[11]^ In both cases, the heterocyclizations proceeded by electrophilic spirocyclization followed by C−C bond migration (Scheme 1, *path A*). Thus, they differed significantly from the conventional electrophilic aromatic substitution (S_E_Ar) mechanism as revealed by Yang, Li, and co‐workers in a cobalt catalysis providing dihydro‐2(1*H*)‐quinolinones (Scheme 1, *path B*).^[12]^ Noteworthy, all of these methods involve precious highly optimized catalyst systems, complex reaction conditions, and rather long reaction times.

**Scheme 1:**
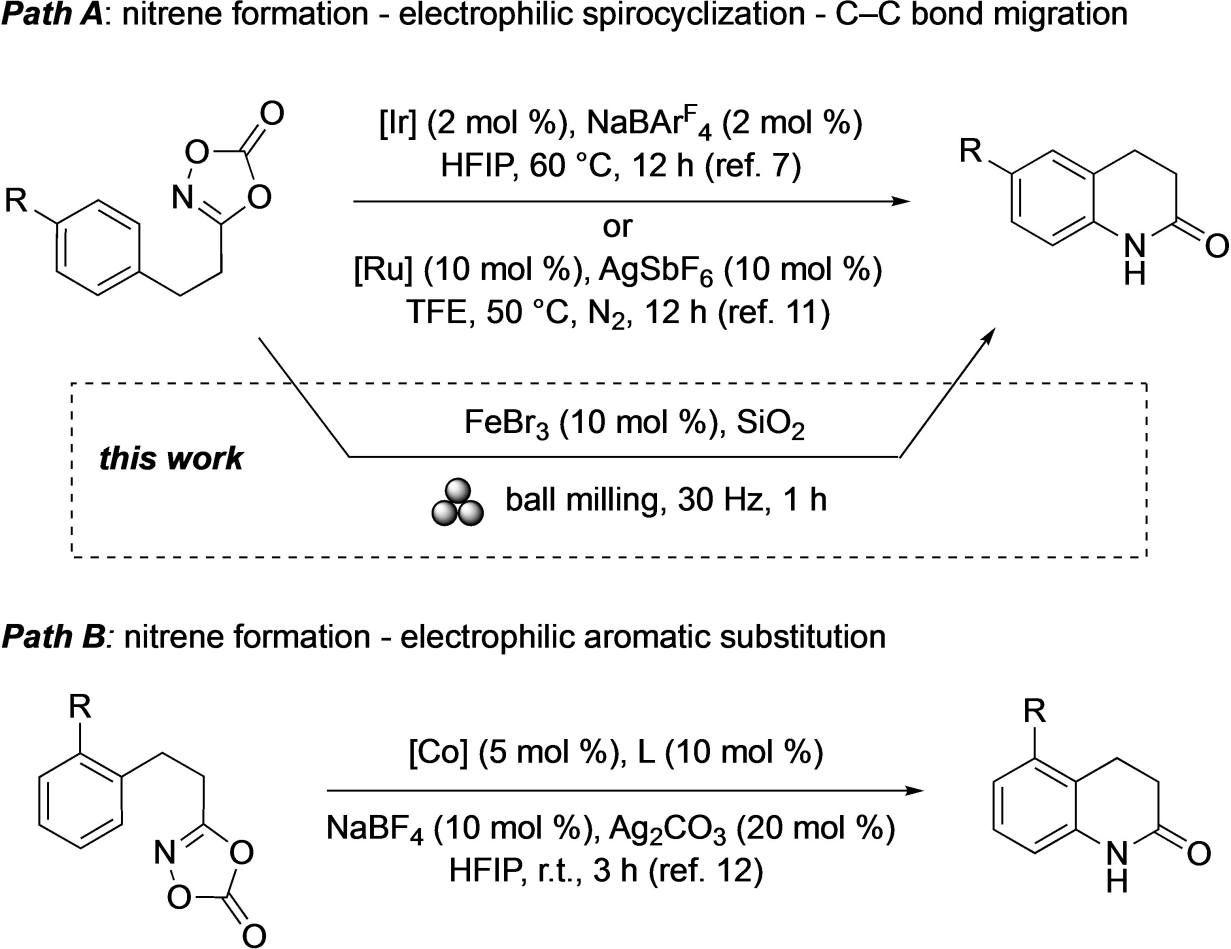
Reported intramolecular amidation reactions and this work.

Recently, mechanochemistry has attracted attention in the organic community due to its board synthetic applicability and its potential to devise benign reaction sequences.^[13]^ In this context, we reported several protocols for directed transition metal‐catalyzed C−H‐bond functionalizations in ball mills.[^14^, ^15^, ^16^] In general, these reactions were solvent‐free, required only low catalyst loadings, and were characterized by simple experimental protocols. We now wondered about fusing the aforementioned amidation procedures leading dihydro‐2(1*H*)‐quinolinones under Ir, Ru, and Co catalysis with our expertise in mechanochemistry. Envisaged was the use of a non‐precious metal catalyst and a simplification of the experimental procedure by performing the reactions under solvent‐free conditions within a short period of time. Here, we report the discovery and successful implementation of a mechanochemical iron catalysis fulfilling such goals.^[17]^

For the initial test reactions, 3‐phenethyl‐1,4,2‐dioxazol‐5‐one (**1 a**) was chosen as representative substrate. Dihydro‐2(1*H*)‐quinolinone **2 a** was the expected product. The initial experiments indicated significant challenges. Thus, milling of **1 a** in the presence of SiO_2_ as grinding agent at a milling frequency of 20 Hz for 1 h gave **2 a** in only trace quantities (Table 1, entry 1). To our surprise, however, the outcome significantly changed when FeCl_2_ was added. Thus, applying 10 mol % of that iron(II) salt led to a remarkable improvement, and **2 a** was obtained in 58 % yield (Table 1, entry 2). The corresponding copper and nickel chlorides did not show this effect (Table 1, entries 3 and 4). The presence of 20 mol % of *rac*‐2,2′‐bis(diphenylphosphino)‐1,1′binaphthyl (*rac*‐BINAP), 1,10‐phenanthroline (1,10‐phen), xphos (2‐dicyclohexylphosphin‐2′,4′,6′‐triisopropylbiphenyl), or dtbpy (4,4′‐di‐*tert‐*butyl‐2.2′‐dipyridyl) in the reaction mixture with FeCl_2_ hampered the catalysis (Table 1, entries 5 and 8). Other iron salts showed similar catalytic effects as FeCl_2_ with FeBr_3_ being superior over all others (Table 1, entries 9 and 12). Thus, with 10 mol % of that iron(III) salt, 62 % of **2 a** were obtained. The yield of **2 a** was further improved by increasing the milling frequency from 20 Hz to 25 Hz and 30 Hz, which gave **2 a** in 70 % and 91 %, respectively (Table 1, entries 13 and 14). Shortening the reaction time from 1 h to 30 min had a negative effect limiting the yield of **2 a** to 57 % (Table 1, entry 15). Thus, the best conditions for the preparation of **2 a** from **1 a** involved milling of the starting material in the presence of 10 mol % of FeBr_3_ and SiO_2_ (300 mg per mmol of **1 a**) at a frequency of 30 Hz for 1 h. Accordingly, **2 a** was obtained in 91 % yield (Table 1, entry 14).^[18]^

**Table 1:** Optimization of the reaction conditions.^[a]^

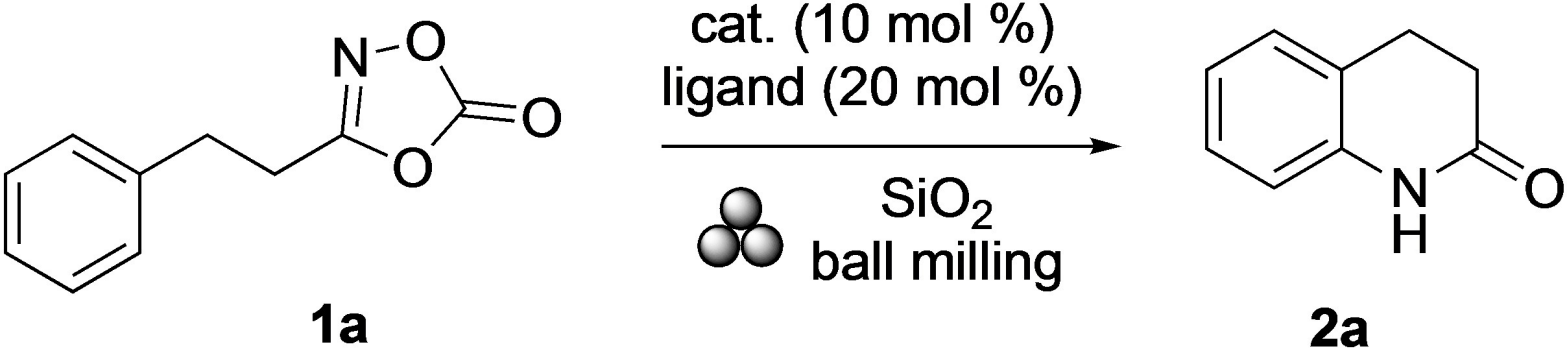					
Entry	Cat.	Ligand	Milling frequency [Hz]	*t* [h]	Yield [%]
1	–	–	20	1	trace
2	FeCl_2_	–	20	1	58
3	CuCl_2_	–	20	1	trace
4	NiCl_2_	–	20	1	trace
5	FeCl_2_	*rac*‐BINAP	20	1	<10
6	FeCl_2_	1,10‐phen	20	1	18
7	FeCl_2_	xphos	20	1	trace
8	FeCl_2_	dtbpy	20	1	<10
9	FeBr_2_	–	20	1	50
10	FeSO_4_	–	20	1	25
11	FeCl_3_	–	20	1	45
12	FeBr_3_	–	20	1	62
13	FeBr_3_	–	25	1	70
**14**	**FeBr_3_**	–	**30**	**1**	**91**
15	FeBr_3_	–	30	0.5	57

[a] Reaction conditions: **1 a** (0.3 mmol), cat. (0.03 mmol), ligand (0.06 mmol) SiO_2_ (90 mg), 10 mL stainless steel milling vessel with one 10 mm stainless steel ball.

Next, the substrate scope was examined (Scheme 2). In the first series of experiments, a number of 3‐arylethyl‐1,4,2‐dioxazol‐5‐ones with *para*‐substituents on the aryl were applied. In all cases, the yields of the corresponding products **2 a**–**g** were high ranging from 74–96 %.^[19]^ Electron‐donating properties of the substituents appeared to be beneficial for the product yields. Thus, the best result was achieved in the conversion of 4‐methoxy‐substituted **1 b**, which led to 3,4‐dihydro‐2(1*H*)‐quinolinone **2 b** in 96 % yield. On the other hand, fluoro‐containing product **2 f** was formed in only 74 % yield. This reactivity pattern was also observed in heterocyclizations of **1 h** and **1 i**, which led to **2 h** and **2 i** in 90 % and 94 % yield, respectively. Only the 4‐methoxy‐substituted aryls had reacted, while none of the alternative products stemming from cyclizations through the other arenes was detected. In all of the aforementioned transformations, the products (**2 a**–**i**) resulted from a sequential electrophilic spirocyclization and C−C bond migration. This mechanistic scenario, which benefited from a stabilization of a developing positive charge by an electron‐donating substituent at an appropriate position (see Scheme 4) was substantiated by the lack of reactivity of 3‐methoxy‐substituted **1 j**. In this case, this stabilization was insufficient, and thus, no product formation occurred. Consequently, **1 j** was recovered in 55 % yield, besides the detection of several unidentified side‐products. Confirming this hypothesis, 3,4‐di(methoxy)‐substituted **1 k** reacted well affording **2 k** in 98 % yield. The reaction outcome with 2‐naphthyl‐containing **1 l** as substrate was complex. In this case, three products (**2 l**, **2 l′a**, and **2 l′b**) were formed. Chromatographically, **2 l** could be separated from a 3 : 1 mixture of **2 l′a**, and **2 l′b**. The product amounts of those two samples corresponded to yields of 21 % and 62 %, respectively. While the formation of **2 l** and **2 l′a** were clearly distinct and mechanistically unique involving an electrophilic spirocyclization/C−C bond migration sequence and an electrophilic aromatic substitution, respectively, **2 l′b** could result from both pathways. The two mechanistic scenarios were also relevant in the reaction of 2‐bromo‐substituted substrate **1 m**, which led to a 5 : 1 mixture of regioisomers **2 m** and **2 m′** in an overall yield of 32 %. Finally, two more β‐disubstituted substrates **1 n** and **1 o** were tested. In both cases, the yields of the corresponding 3,4‐dihydro‐2(1*H*)‐quinolinones **2 n** and **2 o** were high (92 % and 87 %, respectively). For confirming the applicability of the heterocyclization, the reaction of **1 b** was performed on a 1 mmol scale affording **2 b** in 93 % yield.

**Scheme 2:**
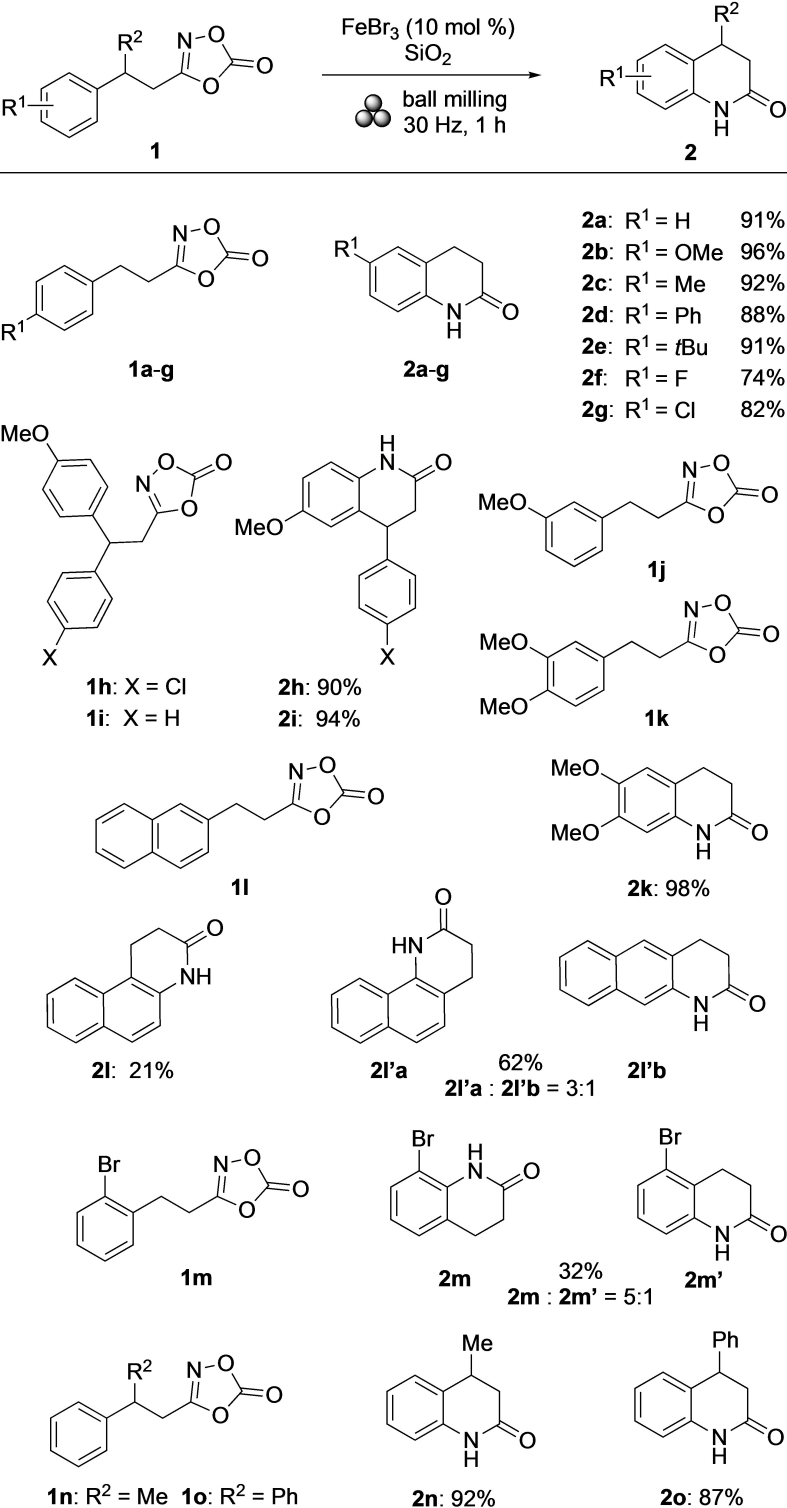
Substrate scope (0.3 mmol scale).

The following two test reactions aimed at gaining mechanistic insight: First, the conversion of **1 b** to **2 b** was evaluated in the presence of 3 equiv of (2,2,6,6‐tetramethylpiperidine‐1‐yl)oxyl (TEMPO). The yield of <10 % of **2 b** (as compared to the previously determined 96 % yield for the same product under standard reaction conditions) suggested the involvement of radicals as relevant intermediates. Second, applying phenolic substrate **1 p** to the heterocyclization conditions gave spiro product **3** in 78 % yield (Scheme 3), making it most likely that also in transformations of other substrates such spiro intermediates could be involved and be of importance for the final product formation.

**Scheme 3:**
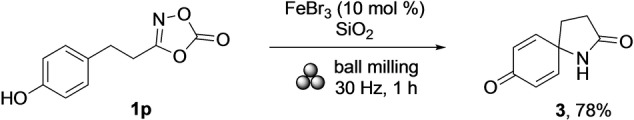
Reaction of **1 p** to give spiro compound **3**.

Based on these observations and in light of results reported by others,^[20]^ a mechanistic pathway can be devised (Scheme 4). Due to experimental challenges of analyzing the reaction progress under mechanochemical conditions, several steps have to remain descriptive at this stage. In analogy to the discussion by Bao, Yu, and co‐workers for reactions (in solution) with a 3‐phenyl substituted dioxazolone and FeCl_3_,^[20b]^ we propose an activation of substrate **1** by coordination of FeBr_3_. Loss of CO_2_ leads to a new species **A**, formally corresponding to an acylnitrene iron intermediate.^[21]^ Although the precise nature of **A** is unknown, we assume that it has a partial radical character as indicated by the hampered product formation after addition of TEMPO. The reactivity of **A** is affected by the substituent R. If the electronic properties of R increase the electron density of the arene and the position of R stabilizes a developing positive charge, as, for example, in **1 b** bearing a methoxy group in *para* position, sequential spirocyclization and C−C‐bond migration occurs leading to the observed 3,4‐dihydro‐2(1*H*)‐quinolinones **2** (here, **2 b**) via intermediates **B** and **C**.^[22]^ The formation of **3** from **1 p** (Scheme 3) supports this proposed reaction path, which is also in accord with the pathways discussed in the literature.[^7^, ^8^, ^11^, ^12^, ^20^] In case the R substituent in **1** does not provide enough of the aforementioned electronic stabilization, electrophilic aromatic substitution (S_E_Ar) reactions or direct nitrene‐type C−H bond insertions can become relevant explaining the formations of products **2 l′b** and **2 m′**.

**Scheme 4:**
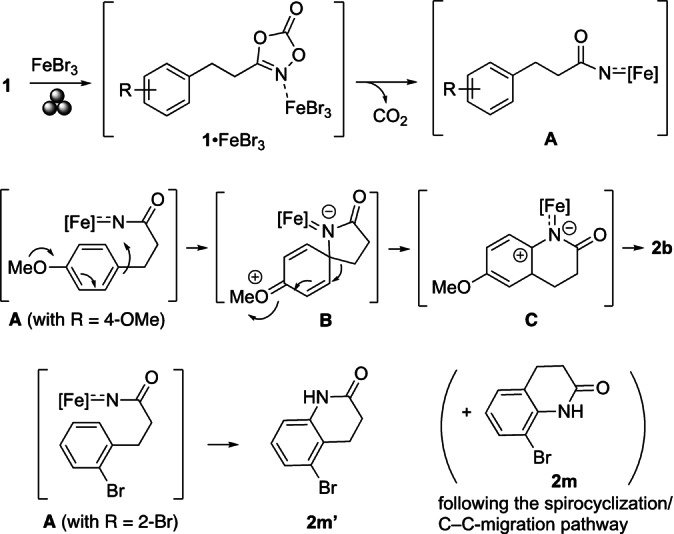
Possible mechanistic pathways.

In summary, we developed a mechanochemical iron catalysis for intramolecular amidation reactions of dioxazolones leading to 3,4‐dihydro‐2(1*H*)‐quinolinones in good to very high yields. No ligand is required for the activation of FeBr_3_, which is applied in a 10 mol % quantity. The reactions are solvent‐free, and the processes are easy to perform in a standard commercial ball mill. Mechanistically, electrophilic spirocyclization/C−C migration sequencies and S_E_Ar reactions leading to C−H‐functionalizations explain the product formation.

## Conflict of interest

The authors declare no conflict of interest.

## Supporting information

As a service to our authors and readers, this journal provides supporting information supplied by the authors. Such materials are peer reviewed and may be re‐organized for online delivery, but are not copy‐edited or typeset. Technical support issues arising from supporting information (other than missing files) should be addressed to the authors.

Supporting Information

## Data Availability

The data that support the findings of this study are available in the Supporting Information of this article.
