# Supplementary material for: Iron‐Catalyzed Intramolecular Arene C(sp2)−H Amidations under Mechanochemical Conditions
Source: Angew Chem Int Ed Engl. 2022 Jun 10;61(30):e202204874. doi: 10.1002/anie.202204874 (PMC9401578; doi:10.1002/anie.202204874)

Angewandte Chemie
Eine Zeitschrift der Gesellschaft Deutscher Chemiker

Supporting Information

Iron-Catalyzed Intramolecular Arene C(sp^2)–H Amidations under Mechanochemical Conditions

*P. Shi, Y. Tu, D. Kong, P. Wu, D. Ma, C. Bolm**

Supporting Information

Table of Content

1. General information S2

2. General procedure for the preparation of 2,3-diaryl-substituted carboxylic acids[S1] S2

3. Syntheses of 3-substituted 1,4,2-dioxazol-5-ones **1** S3

4. General procedure for the syntheses of dihydroquinolin-2-ones **2** S3

5. Scale-up experiment S4

6. References S4

7. Characterizing data S5

8. NMR spectra S11

1. General information

Unless otherwise noted, the materials were purchased from commercial suppliers and used without further purification. All the solvents were treated according to general methods. The reactions were monitored by thin layer chromatography (TLC) with aluminium sheets silica gel 60 F254 from Merck, and Column chromatography purifications were performed using 200–300 mesh silica gel. ^{1}H and ^{13}C NMR spectra were recorded with an Agilent VNMRS 600, Agilent VNMRS 400 or Varian Mercury 300 in deuterated solvents. Chemical shifts (δ) are reported in parts per million (ppm) and spin-spin coupling constants (*J*) are given in Hz, Multiplicities are recorded as: s = singlet, d = doublet, t = triplet, q = quartet, dd = doublet of doublets, m = multiplet. The IR spectra were recorded with a PerkinElmer Spectrum 100 spectrometer with an attached UATR device Diamond KRS-5. All IR data were collected by attenuated total reflectance (ATR) and wavenumbers ν are given in cm^{-1}. Mass spectra were recorded with a Finnigan SSQ Finnigan 7000 spectrometer (EI, 70 eV). High resolution mass spectra (HRMS) were recorded on a Thermo Scientific LTQ Orbitrap XL spectrometer. Melting points (mp) were determined on a Büchi B-540 melting point apparatus.

2. General procedure for the preparation of 3,3-diaryl-substituted carboxylic acid.[S1]

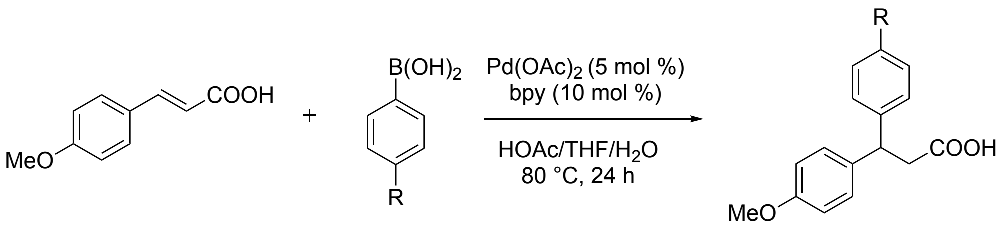

4-Methoxycinnamic acid (178.19 mg, 1.0 mmol), the arylboronic acid (2.0 mmol), $Pd(OAc)_2$ (11.2 mg, 0.05 mmol), bpy (15.6 mg, 0.1 mmol), HOAc (1.0 mL), THF (2.0 mL), and H_2O (0.6 mL) were added to a 10 mL flask and stirred at 80 °C in an oil bath under an air atmosphere for 24 h. Then, the solvents were removed under vacuum. Subsequently, water (5 mL) was added to the flask, and the mixture was stirred at 60 °C in an oil bath for 10 min. After cooling to ambient temperature, the solution was extracted with CH_2Cl_2 (3 × 10 mL), dried over anhydrous Na_2SO_4, and concentrated in vacuo. Purification by silica gel flash chromatography (EA/PE = 1/2) gave the 3,3-diaryl substituted carboxylic acid.

3. Syntheses of 3-substituted-1,4,2-dioxazol-5-ones 1

3.1. Synthesis of hydroxamic acids.[S2]

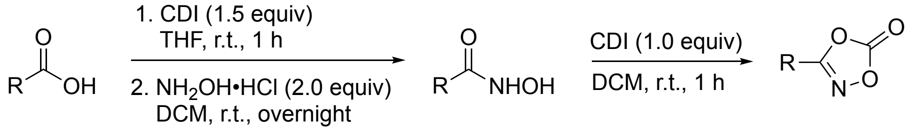

1,1'-Carbonyldiimidazole (CDI, 1.5 equiv) was added to the respective carboxylic acid (1.0 equiv) in dry tetrahydrofuran (THF, 1.0 M) at room temperature. The reaction mixture was stirred for 1-2 h. Then, powdered hydroxylamine hydrochloride (2.0 equiv) was added. The resulting mixture was stirred overnight, and then diluted with 5% aq. $KHSO_4$ and extracted with ethyl acetate. The combined organic layer was washed with water and brine, dried over anhydrous Na_2SO_4, and concentrated in vacuo. Purification by silica gel flash chromatography (DCM/methanol = 30/1–10/1) gave the hydroxamic acid.

3.2 Syntheses of 3-substituted-1,4,2-dioxazol-5-ones[S3]

To a stirred solution of the hydoxamic acid (5.0 mmol) in dichloromethane (50 mL) was added 1,1’-carbonyldiimidazole (0.81 g, 5.0 mmol) in one portion at room temperature. After stirring for 30 min, the reaction mixture was quenched with 1 N HCl (30 mL), extracted with dichloromethane (3 x 50 mL) and dried over magnesium sulfate. The solvent was removed under reduced pressure to afford 3-substituted 1,4,2-dioxazol-5-one **1**.

4. General procedure for the syntheses of dihydroquinolin-2-ones 2

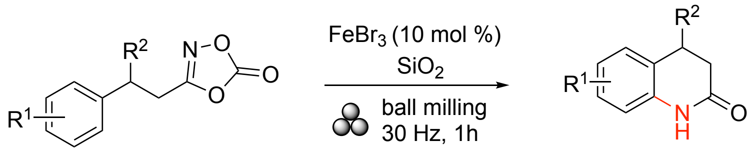

1 2

A mixture of **1** (0.3 mmol), $FeBr_3$ (8.9 mg, 0.03 mmol), and SiO_2 (90 mg) was added in a 10 mL stainless steel milling vessel with one 10 mm stainless steel ball and reacted at 30 Hz for 1 h. Then, the crude product was transferred to a 25 mL flask by washing with DCM (3 x 5 mL). Purification by column chromatography (EA/PE = 1/5–1/3) on silica gave product **2**.

Note: The removal of the product from the milling vessel can also be done with ethyl acetate instead of DCM, which in a tested system led to the same result.

5. Scale-up experiment

As described in the general procedure (section 4) using, **1b** (221 mg, 1.0 mmol), $FeBr_3$ (29.7 mg, 0.1 mmol), and SiO_2 (180 mg) were added in a 5 mL stainless steel milling vessel with one 10 mm stainless steel ball and reacted at 30 Hz for 1 h. Yield: 164.5 mg of **2b** (93%).

6. References

[S1] R. Liu, Z. Yang, Y. Ni, K. Song, K. Shen, S. Lin, Q. Pan, *J. Org. Chem.* **2017**, *82*, 8023-8030.

[S2] H. Wang, Y. Park, Z. Bai, S. Chang, G. He, G. Chen, *J. Am. Chem. Soc.* **2019**, *141*, 7194-7201.

[S3] Q. Xing, C. M. Chan, Y. W. Yeung, W. Y. Yu, *J. Am. Chem. Soc.* **2019**, *141*, 3849-3853.

7. Characterizing data

3,4-Dihydroquinolin-2(1*H*)-one (2a)

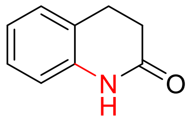

Following the general procedure afforded the product as a white solid (41.1 mg, 91% yield); mp = 161.7-163.5 °C. IR (ATR): ν = 3185, 3081, 2916, 2856, 1905, 1785, 1674, 1592, 1489, 1431, 1380, 1336, 1278, 1244, 1197, 1031, 912, 811, 742, 678. ^{1}H NMR (600 MHz, $CDCl_3$) δ 9.22 (s, 1H), 7.16 (dd, *J* = 13.4, 7.2 Hz, 2H), 6.98 (t, *J* = 7.4 Hz, 1H), 6.85 (d, *J* = 7.5 Hz, 1H), 2.97 (t, *J* = 7.5 Hz, 2H), 2.65 (m, 2H). ^{13}C{^{1}H} NMR (150 MHz, $CDCl_3$) δ 172.3, 137.4, 127.9, 127.5, 123.6, 123.1, 115.6, 30.8, 25.4. MS (EI): *m/z* (%) = 181.0, 148.1, 147.1, 119.1, 118.1, 91.1. HRMS *m/z*: $[M+Na]^+$ calcd for C_9H_9ONNa: 170.0576, found:170.0579.

6-Methoxy-3,4-dihydroquinolin-2(1*H*)-one (2b)

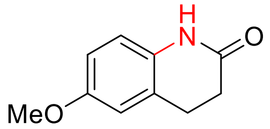

Following the general procedure afforded the product as a white solid (51.0 mg, 96% yield); mp = 138.5-140.2 °C. IR (ATR): ν = 3192, 3059, 3002, 2924, 2856, 1727, 1660, 1499, 1428, 1384, 1275, 1239, 1197, 1124, 1036, 880, 825, 792, 707. ^{1}H NMR (600 MHz, $CDCl_3$) δ 7.97 (s, 1H), 6.86 – 6.39 (m, 3H), 3.78 (s, 3H), 2.94 (t, *J* = 7.5 Hz, 2H), 2.73 – 2.53 (m, 2H). ^{13}C{^{1}H} NMR (150 MHz, $CDCl_3$) δ 171.2, 155.7, 130.7, 125.1, 116.1, 113.9, 112.5, 55.6, 30.6, 25.8. MS (EI): *m/z* (%) = 293.1, 178.1, 177.1, 162.1, 149.1, 134.1, 121.1, 78.2. HRMS *m/z*: $[M+Na]^+$ calcd for $C_{10}H_{11}NO_2Na$: 200.0682, found: 200.0683.

6-Methyl-3,4-dihydroquinolin-2(1*H*)-one (2c)

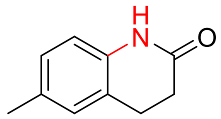

Following the general procedure afforded the product as a white solid (44.4 mg, 92% yield); mp = 126.0-127.9 °C. IR (ATR): ν = 3193, 3057, 2923, 2856, 2743, 2117, 1668, 1614, 1502, 1375, 1252, 1192, 1126, 867, 813, 773, 705. ^{1}H NMR (600 MHz, $CDCl_3$) δ 8.70 (s, 1H), 6.97 (s, 2H), 6.79 – 6.56 (m, 1H), 2.93 (t, *J* = 7.5 Hz, 2H), 2.76 – 2.47 (m, 2H), 2.29 (s, 3H). ^{13}C{^{1}H} (150 MHz, $CDCl_3$) δ 171.9, 134.8, 132.6, 128.6, 127.9, 123.5, 115.3, 30.8, 25.4, 20.8. MS (EI): *m/z* (%) = 239.0, 162.1, 161.1, 133.1, 132.1, 106.1, 91.1. HRMS *m/z*: $[M+Na]^+$ calcd for $C_{10}H_{11}NONa$: 184.0733, found: 184.0735.

6-Phenyl-3,4-dihydroquinolin-2(1*H*)-one (2d)

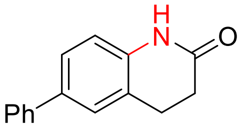

Following the general procedure afforded the product as a white solid (58.9 mg, 88% yield); mp = 186.7-188.4 °C. IR (ATR): ν =3187, 3124, 3060, 2957, 2922, 2851, 1897, 1708, 1658, 1480, 1367, 1330, 1210, 1179, 915, 890, 835, 796, 756. ^{1}H NMR (600 MHz, $CDCl_3$) δ 8.55 (s, 1H), 7.55 (d, *J* = 7.4 Hz, 2H), 7.42 (dd, *J* = 14.5, 7.3 Hz, 4H), 7.33 (t, *J* = 7.4 Hz, 1H), 6.88 (d, *J* = 7.8 Hz, 1H), 3.04 (t, *J* = 7.5 Hz, 2H), 2.69 (t, *J* = 7.5 Hz, 2H). ^{13}C{^{1}H} (150 MHz, $CDCl_3$) δ 171.7, 140.5, 136.6, 136.3, 128.8, 128.7, 127.1, 126.8, 126.3, 124.1, 115.8, 30.8, 25.5. MS (EI): *m/z* (%) =224.1, 223.1, 195.1, 194.1, 180.1, 167.1, 165.0, 152.1. HRMS *m/z*: $[M+Na]^+$ calcd for $C_{15}H_{13}NONa$: 246.0889, found: 246.0892.

6-(*Tert*-butyl)-3,4-dihydroquinolin-2(1*H*)-one (2e)

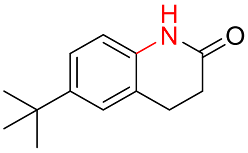

Following the general procedure afforded the product as a white solid (55.5 mg, 91% yield); mp = 153.2-155.6 °C. IR (ATR): ν =3188, 3067, 2951, 1868, 1832, 1684, 1597, 1507, 1372, 1194, 817, 759, 699. ^{1}H NMR (600 MHz, $CDCl_3$) δ 8.15 (s, 1H), 7.19 (d, *J* = 10.4 Hz, 2H), 6.71 (d, *J* = 8.1 Hz, 1H), 2.96 (t, *J* = 7.5 Hz, 2H), 2.73 – 2.51 (m, 2H), 1.30 (s, 9H). ^{13}C{^{1}H} (150 MHz, $CDCl_3$) δ 171.6, 146.2, 134.7, 125.0, 124.3, 123.2, 114.9, 34.3, 31.4, 30.9, 25.7. MS (EI): *m/z* (%) = 204.2, 203.1, 189.1, 188.1, 160.1, 146.1, 91.1. HRMS *m/z*: $[M+Na]^+$ calcd for $C_{13}H_{17}NONa$: 226.1202, found: 226.1201.

6-Fluoro-3,4-dihydroquinolin-2(1*H*)-one (2f)

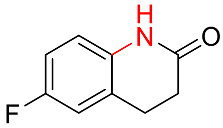

Following the general procedure afforded the product as a white solid (36.6 mg, 74% yield); mp = 157.8-159.2 °C. IR (ATR): ν =3199, 3064, 2920, 2855, 2751, 1885, 1668, 1498, 1383, 1225, 1140, 1108, 944, 868, 811. ^{1}H NMR (600 MHz, $CDCl_3$) δ 8.13 (s, 1H), 6.88 (dd, *J* = 12.9, 8.3 Hz, 2H), 6.71 (dd, *J* = 8.4, 4.7 Hz, 1H), 2.96 (t, *J* = 7.5 Hz, 2H), 2.63 (t, *J* = 7.5 Hz, 2H). ^{19}F NMR (565 MHz, $CDCl_3$) δ -120.14. ^{13}C{^{1}H} (150 MHz, $CDCl_3$) δ 171.3, 158.7 (d, *J* = 241.8 Hz), 133.4, 125.5 (d, *J* = 8.1 Hz), 116.3 (d, *J* = 8.4 Hz), 115.0 (d, *J* = 23.1 Hz), 114.1 (d, *J* = 22.7 Hz), 30.3, 25.5.MS (EI): *m/z* (%) = 166.1, 165.0, 137.1, 136.1, 122.1, 110.1, 109.1, 96.1. HRMS *m/z*: $[M+Na]^+$ calcd for C_9H_8NOFNa: 188.0482, found: 188.0483.

6-Chloro-3,4-dihydroquinolin-2(1*H*)-one (2g)

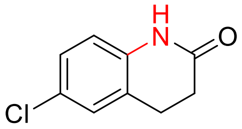

Following the general procedure afforded the product as a white solid (44.5 mg, 82% yield); mp = 172.3-173.8 °C. IR (ATR): ν =3198, 3079, 2924, 1867, 1831, 1675, 1590, 1489, 1378, 1188, 1086, 927, 813, 764. ^{1}H NMR (600 MHz, $CDCl_3$) δ 8.12 (s, 1H), 7.15 (dd, *J* = 10.9, 2.6 Hz, 2H), 6.70 (d, *J* = 8.2 Hz, 1H), 3.25 – 2.82 (m, 2H), 2.77 – 2.39 (m, 2H). $^{13}C\{^{1}H\}$ (150 MHz, $CDCl_3$) δ 171.1, 135.8, 128.1, 127.5, 125.4, 116.4, 30.3, 25.3. MS (EI): *m/z* (%) = 293.1, 183.0, 181.0, 153.0, 152.0, 125.0, 117.1, 91.1. HRMS *m/z*: $[M+H]^+$ calcd for $C_9H_8NOClNa$: 204.0187, found: 204.0187.

4-(4-Chlorophenyl)-6-methoxy-3,4-dihydroquinolin-2(1*H*)-one (2h)

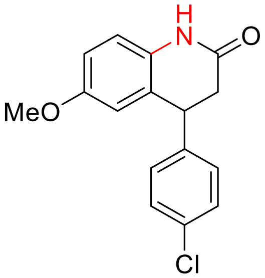

Following the general procedure afforded the product as a white solid (77.5 mg, 90% yield). mp = 174.4-175.6 °C. IR (ATR): ν =3187, 3055, 2956, 2742, 1768, 1667, 1493, 1379, 1226, 1163, 1034, 828, 744, 718. ^{1}H NMR (600 MHz, $CDCl_3$) δ 9.51 (s, 1H), 7.31 – 7.27 (m, 2H), 7.12 (d, *J* = 8.4 Hz, 2H), 6.85 (d, *J* = 8.6 Hz, 1H), 6.75 (dd, *J* = 8.6, 2.7 Hz, 1H), 6.46 (d, *J* = 2.6 Hz, 1H), 4.51 – 4.00 (m, 1H), 3.70 (s, 3H), 2.91 (dd, *J* = 16.1, 6.2 Hz, 1H), 2.83 (dd, *J* = 16.1, 8.3 Hz, 1H). $^{13}C\{^{1}H\}$ NMR (150 MHz, $CDCl_3$) δ 170.6, 155.9, 139.9, 133.1, 130.7, 129.2, 129.1, 127.4, 116.9, 114.3, 113.0, 55.6, 41.7, 38.3. MS (EI): *m/z* (%) = 289.0, 287.0, 272.0, 258.0, 244.0, 209.1, 201.0, 176.1, 167.1, 149.0, 90.1. HRMS *m/z*: $[M+Na]^+$ calcd for $C_{16}H_{14}NO_2ClNa$: 310.0605, found: 310.0597.

6-Methoxy-4-phenyl-3,4-dihydroquinolin-2(1*H*)-one (2i)

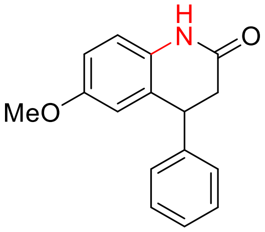

Following the general procedure afforded the product as a white solid (71.4 mg, 94% yield); mp = 149.6-151.3 °C. IR (ATR): ν =3196, 3060, 2928, 1667, 1602, 1494, 1458, 1376, 1292, 1248, 1217, 1150, 1115, 1035, 822, 790, 771, 701. ^{1}H NMR (600 MHz, $CDCl_3$) δ 8.96 (s, 1H), 7.13 (t, *J* = 7.5 Hz, 2H), 7.06 (dd, *J* = 9.0, 5.6 Hz, 1H), 6.99 (d, *J* = 7.3 Hz, 2H), 6.63 (d, *J* = 8.6 Hz, 1H), 6.54 (dd, *J* = 8.6, 2.7 Hz, 1H), 6.28 (d, *J* =

2.5 Hz, 1H), 4.34 – 3.92 (m, 1H), 3.48 (s, 3H), 2.90 – 2.47 (m, 2H). $^{13}C\{^1H\}$ NMR (150 MHz, $CDCl_3$) δ 170.7, 155.8, 141.4, 130.7, 129.0, 128.1, 127.8, 127.3, 116.6, 114.4, 112.8, 55.5, 42.3, 38.4. MS (EI): *m/z* (%) = 291.1, 254.1, 253.1, 238.1, 225.1, 224.1, 210.1, 167.1, 165.1, 91.1. HRMS *m/z*: $[M+Na]^+$ calcd for $C_{16}H_{15}NO_2Na$: 276.0995, found: 276.0988.

6,7-Dimethoxy-3,4-dihydroquinolin-2(1*H*)-one (2k)

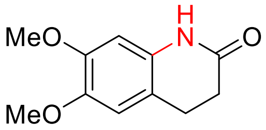

Following the general procedure afforded the product as a white solid (60.9 mg, 98% yield); mp = 135.6-136.4 °C. IR (ATR): ν =3190, 3083, 2988, 2925, 2852, 1693, 1620, 1515, 1461, 1380, 1231, 1190, 1008, 845, 795, 762, 730. ^1H NMR (600 MHz, $CDCl_3$) δ 8.86 (s, 1H), 6.68 (s, 1H), 6.39 (s, 1H), 3.85 (s, 3H), 3.84 (s, 3H), 2.89 (t, *J* = 7.6 Hz, 2H), 2.72 – 2.37 (m, 2H). $^{13}C\{^1H\}$ NMR (150 MHz, $CDCl_3$) δ 171.9, 148.5, 144.8, 130.7, 114.9, 111.7, 100.5, 56.4, 56.2, 31.0, 25.1. MS (EI): *m/z* (%) = 208.1, 207.1, 192.1, 164.1, 149.0, 122.1, 93.1. HRMS *m/z*: [M+Na]+ calcd for $C_{11}H_{13}NO_3Na$: 230.0788, found: 230.0789.

1,4-Dihydrobenzo[f]quinolin-3(2*H*)-one (2l)

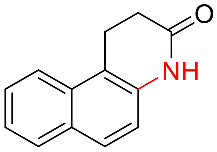

Following the general procedure and purification by column chromatography (EA/PE = 1/6–1/4) on silica allowed to separate product **2l** from a mixture of **2l'a** : **2l'b**. Accordingly, **2l** was obtained as a white solid (12.4 mg, 21% yield); mp = 225.8-227.6 °C. IR (ATR): ν =3215, 3114, 2927, 2855, 2109, 1668, 1575, 1467, 1381, 1333, 1267, 1184, 1156, 991, 752, 670. ^1H NMR (600 MHz, $CDCl_3$) δ 7.95 (s, 1H), 7.90 (d, *J* = 8.5 Hz, 1H), 7.81 (d, *J* = 8.1 Hz, 1H), 7.72 (d, *J* = 8.6 Hz, 1H), 7.62 – 7.48 (m, 1H), 7.40 (t, *J* = 7.5 Hz, 1H), 6.97 (d, *J* = 8.6 Hz, 1H), 3.36 (t, *J* = 7.8 Hz, 2H), 2.82 – 2.77 (m, 2H). $^{13}C\{^1H\}$ NMR (150 MHz, $CDCl_3$) δ 171.1, 134.4, 131.5, 130.5, 128.8, 128.3, 127.1, 124.3, 122.6, 116.5, 116.2, 30.4, 21.1. MS (EI): *m/z* (%) = 278.0, 276.9, 198.1, 197.1, 169.1, 168.1, 154.1, 84.7. HRMS *m/z*: $[M+Na]^+$ calcd for $C_{13}H_{11}NONa$: 220.0733, found: 220.0734.

3,4-Dihydrobenzo[*h*]quinolin-2(1*H*)-one (2l'a) and 3,4-dihydrobenzo[*g*]quinolin-2(1*H*)-one (2l'b)

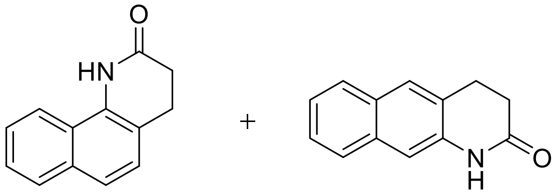

Following the general procedure afforded the product as a white solid (36.7 mg, 62% yield, **2l'a** : **2l'b** = 3 : 1); mp = 228.9-230.3 °C. IR (ATR): ν =3220, 3118, 2930, 2850, 2327, 1670, 1574, 1468, 1385, 1335, 1269, 1246, 1184, 993, 815, 789, 754. ^{1}H NMR (600 MHz, $CDCl_3$) δ 9.27 (s, 0.3H), 8.99 (s, 1H), 8.31 – 8.17 (m, 0.3H), 8.05 – 7.98 (m, 0.3H), 7.94 (d, *J* = 8.5 Hz, 1H), 7.84 (d, *J* = 8.2 Hz, 1H), 7.61 (m, 1.2H), 7.55 (ddd, *J* = 23.0, 10.8, 4.7 Hz, 2H), 7.49 (t, *J* = 7.2 Hz, 1H), 7.30 (d, *J* = 8.3 Hz, 1H), 3.40 – 3.03 (m, 2.7H), 2.87 – 2.46 (m, 2.7H). ^{13}C{^{1}H} NMR (150 MHz, $CDCl_3$) δ 172.1, 171.9, 133.1, 132.2, 132.0, 131.2, 129.8, 128.7, 128.0, 127.2, 127.2, 126.5, 126.1, 125.8, 123.6, 123.0, 122.4, 120.3, 120.0, 119.5, 119.3, 116.3, 31.0, 30.8, 26.0, 25.7. MS (EI): *m/z* (%) = 277.9, 274.9, 198.1, 197.1, 169.1, 168.1, 154.0, 115.0. HRMS *m/z*: $[M+Na]^+$ calcd for $C_{13}H_{11}NONa$: 220.0733, found: 220.0726.

8-Bromo-3,4-dihydroquinolin-2(1H)-one (2m) and 5-bromo-3,4-dihydroquinolin-2(1H)-one (2m')

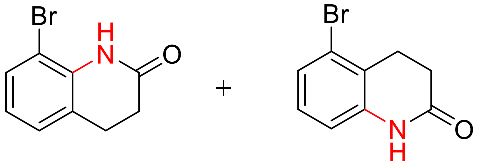

Following the general procedure afforded the product as a white solid (21.6 mg, 32% yield, **2m** : **2m'** = 5 : 1); mp = 182.1-184.4 °C. IR (ATR): ν =3237, 2923, 2854, 1673, 1599, 1473, 1367, 1266, 1186, 1135, 943, 791, 756, 715, 686. ^{11}H NMR (600 MHz, $CDCl_3$) δ 7.82 (s, 1H), 7.74 (s, 0.2H), 7.56 (d, *J* = 8.2 Hz, 0.2H), 7.43 (d, *J* = 8.0 Hz, 1H), 7.14 (d, *J* = 7.5 Hz, 1H), 7.11 (d, *J* = 7.3 Hz, 0.2H), 6.95 (t, *J* = 7.8 Hz, 0.2H), 6.89 (t, *J* = 7.8 Hz, 1H), 3.06 – 2.91 (m, 3.6H), 2.77 – 2.57 (m, 3.6H). ^{13}C{^{1}H} NMR (150 MHz, $CDCl_3$) δ 171.8, 170.5, 152.8, 135.2, 131.1, 130.9, 127.7, 127.2, 126.5, 125.6, 123.8, 123.3, 109.6, 107.7, 30.6, 29.7, 26.0, 25.8. MS (EI): *m/z* (%) = 294.1, 293.1, 225.0, 197.0, 167.0, 149.0, 127.1, 71.2. HRMS *m/z*: $[M+Na]^+$ calcd for $C_9H_8NOBrNa$: 247.9682, found: 247.9681.

4-Methyl-3,4-dihydroquinolin-2(1H)-one (3n)

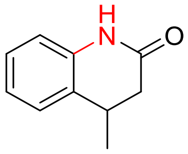

Following the general procedure afforded the product as a white solid (44.5 mg, 92% yield); mp = 113.1-115.4 °C. IR (ATR): 3196, 3072, 2962, 2920, 1670, 1588, 1485,

1375, 1250, 1181, 864, 819, 748, 663. ^{1}H NMR (600 MHz, $CDCl_3$) δ 8.54 (s, 1H), 7.19 (dd, *J* = 15.6, 7.7 Hz, 2H), 7.02 (t, *J* = 7.5 Hz, 1H), 6.81 (d, *J* = 7.8 Hz, 1H), 3.14 (dd, *J* = 13.5, 6.8 Hz, 1H), 2.74 (dd, *J* = 16.1, 5.8 Hz, 1H), 2.43 (dd, *J* = 16.1, 7.2 Hz, 1H), 1.31 (d, *J* = 7.0 Hz, 3H). ^{13}C{^{1}H} NMR (101 MHz, $CDCl_3$) δ 171.3, 136.4, 128.8, 127.5, 126.6, 123.4, 115.6, 77.2, 77.0, 76.8, 38.4, 30.8, 19.8. MS (EI): *m/z* (%) = 289.1, 162.1, 161.1, 146.1, 128.1, 118.1, 91.1. HRMS *m/z*: $[M+Na]^+$ calcd for $C_{10}H_{11}NONa$: 184.0733, found: 184.0727.

4-Phenyl-3,4-dihydroquinolin-2(1H)-one (2o)

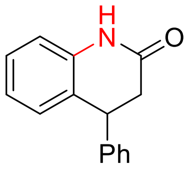

Following the general procedure afforded the product as a white solid (58.2 mg, 87% yield); mp = 142.5-145.3 °C. IR (ATR): ν =3191, 3126, 3057, 2974, 2915, 1664, 1592, 1485, 1383, 1317, 1249, 1184, 835, 756, 703. ^{1}H NMR (600 MHz, $CDCl_3$) δ 9.00 (s, 1H), 7.34 (t, *J* = 7.5 Hz, 2H), 7.30 – 7.26 (m, 1H), 7.24 – 7.17 (m, 3H), 7.02 – 6.83 (m, 3H), 4.68 – 4.01 (m, 1H), 3.43 – 2.61 (m, 2H). ^{13}C{^{1}H} NMR (150 MHz, $CDCl_3$) δ 171.0, 141.5, 137.1, 128.9, 128.4, 128.0, 127.8, 127.3, 126.7, 123.4, 115.8, 42.0, 38.4. MS (EI): *m/z* (%) = 224.1, 223.1, 222.1, 195.1, 194.1, 180.1, 146.0, 118.1, 91.1. HRMS *m/z*: $[M+Na]^+$ calcd for $C_{15}H_{13}NONa$: 246.0889, found: 246.0887.

1-Azaspiro(4.5)deca-6,9-diene-2,8-dione (3)

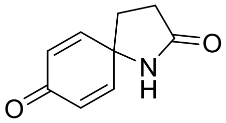

Following the general procedure afforded the product as a white solid (38.1 mg, 78% yield). mp = 168.5-170.4°C. IR (ATR): ν =3246, 3047, 2926, 2859, 2331, 1692, 1656, 1613, 1503, 1405, 1314, 1244, 1204, 1092, 857, 828, 719, 684. ^{1}H NMR (600 MHz, $CDCl_3$) δ 6.83 (d, *J* = 9.2 Hz, 2H), 6.24 (d, *J* = 9.3 Hz, 2H), 6.12 (s, 1H), 2.56 (t, *J* = 8.0 Hz, 2H), 2.27 (t, *J* = 8.1 Hz, 2H). ^{13}C{^{1}H} NMR (150 MHz, $CDCl_3$)δ 184.3, 177.2, 149.2, 128.9, 57.4, 32.3, 29.3. MS (EI): m/z (%) =165.1, 163.0, 149.0, 135.1, 121.1, 108.1, 107.1, 106.1, 91.1, 80.1. HRMS *m/z*: $[M+Na]^+$ calcd for $C_9H_9NO_2Na$:186.0526, found: 186.0520.

8. NMR spectra

^{1}H NMR spectrum of compound 2a (600 MHz, $CDCl_3$)

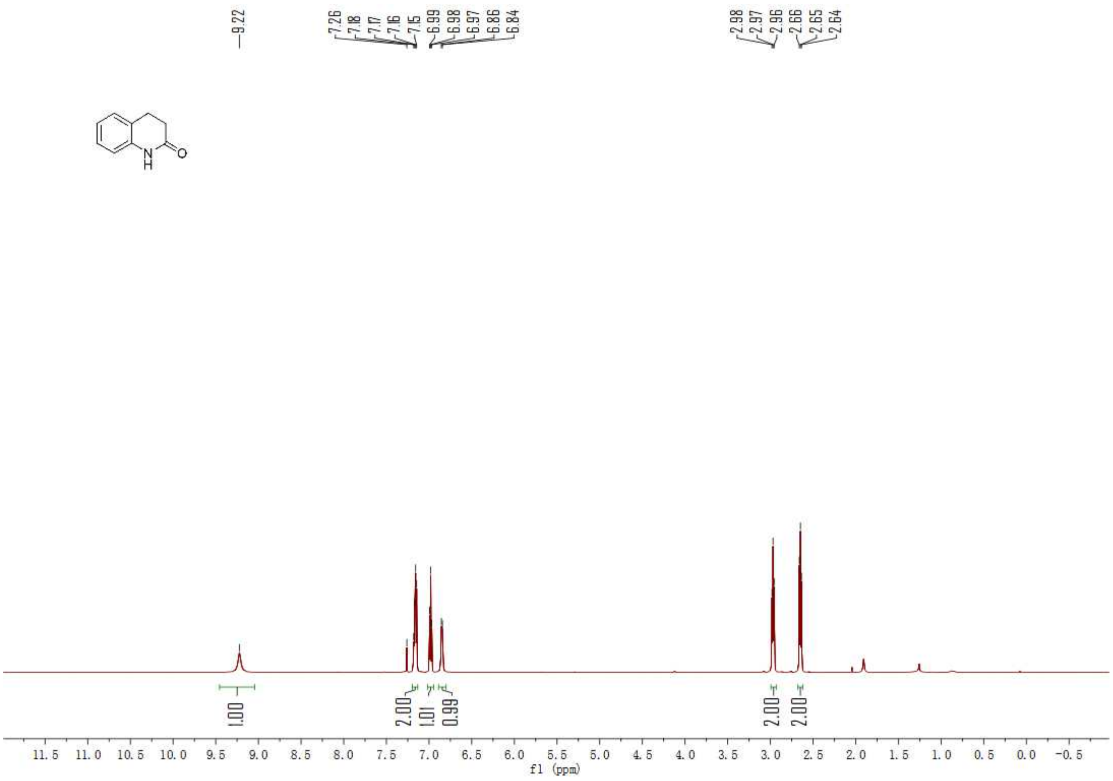

^{13}C{^{1}H} NMR spectrum of compound 2a (150 MHz, $CDCl_3$)

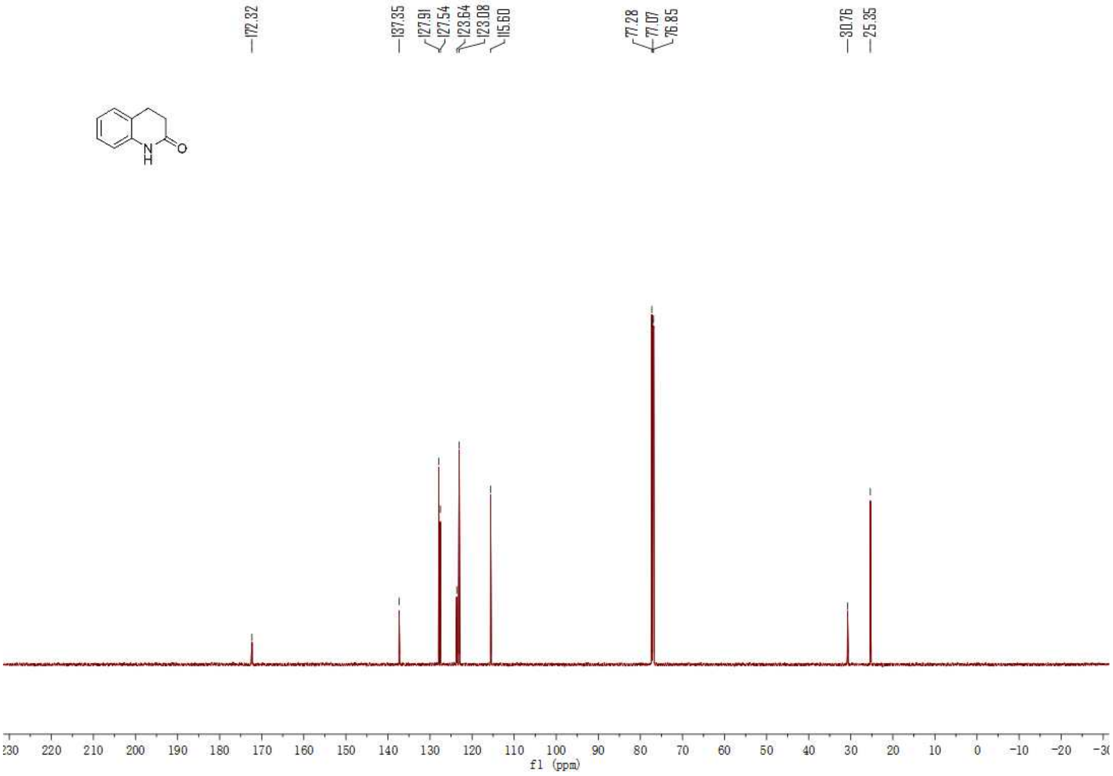

^{1}H NMR spectrum of compound 2b (600 MHz, $CDCl_3$)

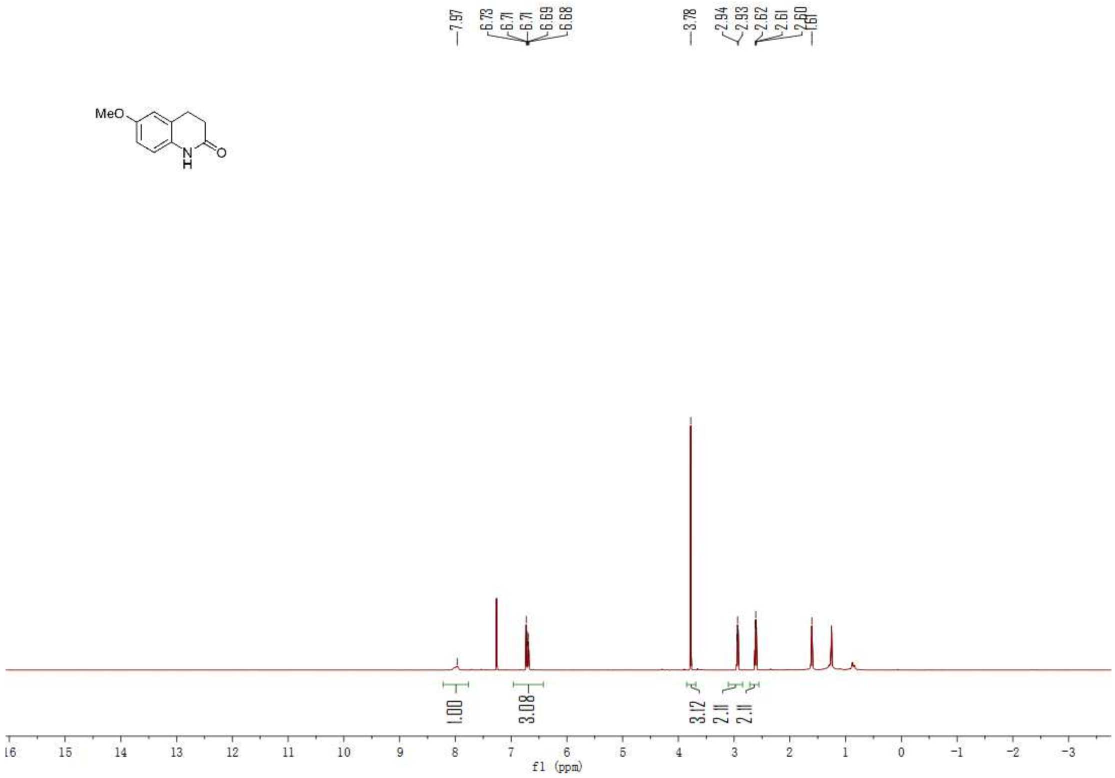

^{13}C{^{1}H} NMR spectrum of compound 2b (150 MHz, $CDCl_3$)

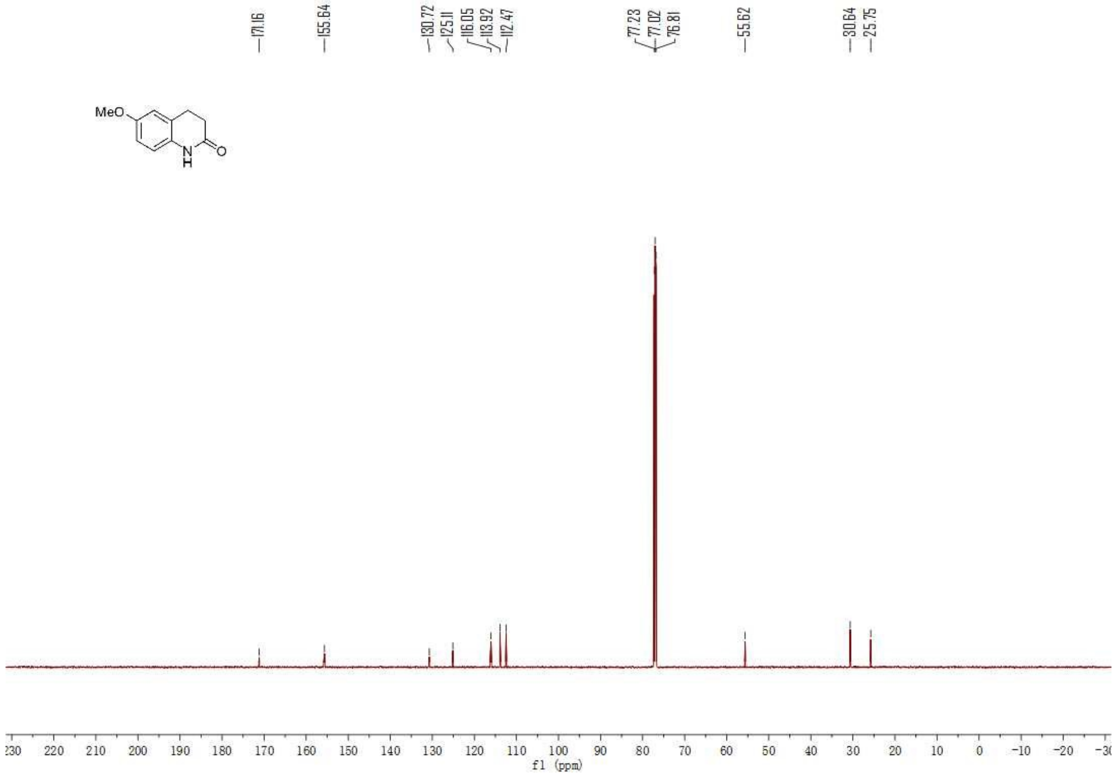

^{1}H NMR spectrum of compound 2c (600 MHz, $CDCl_3$)

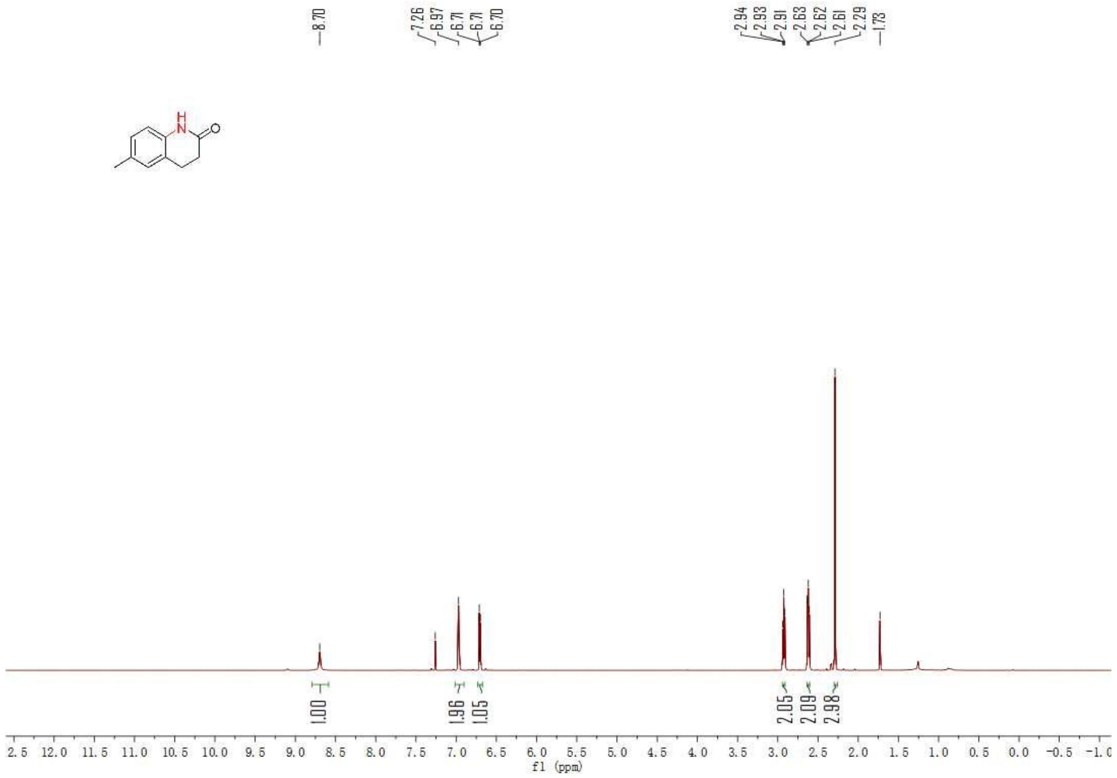

^{13}C{^{1}H} NMR spectrum of compound 2c (150 MHz, $CDCl_3$)

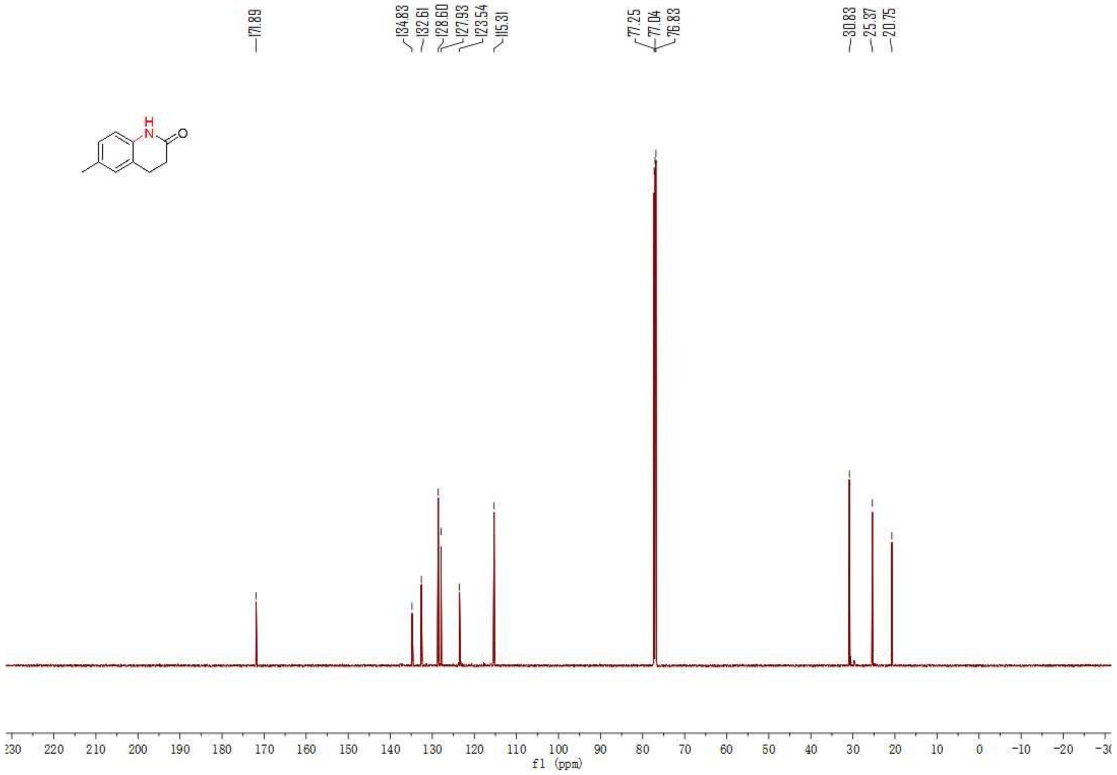

^{1}H NMR spectrum of compound 2d (600 MHz, $CDCl_3$)

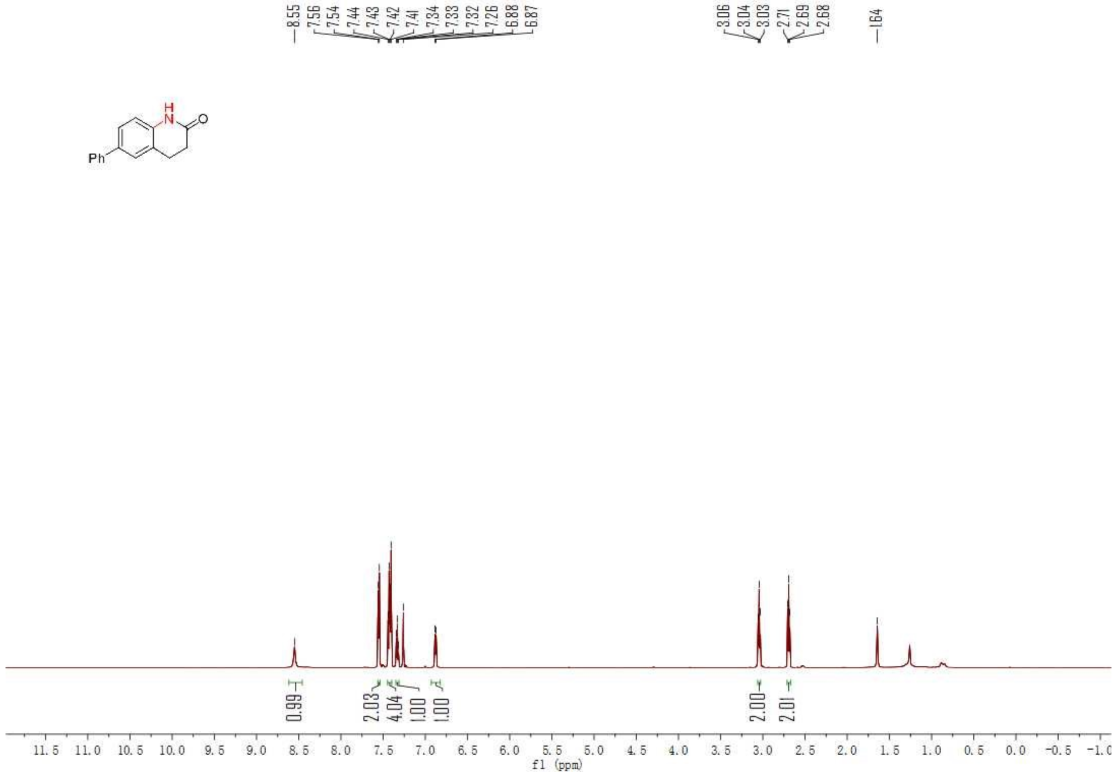

^{13}C{^{1}H} NMR spectrum of compound 2d (150 MHz, $CDCl_3$)

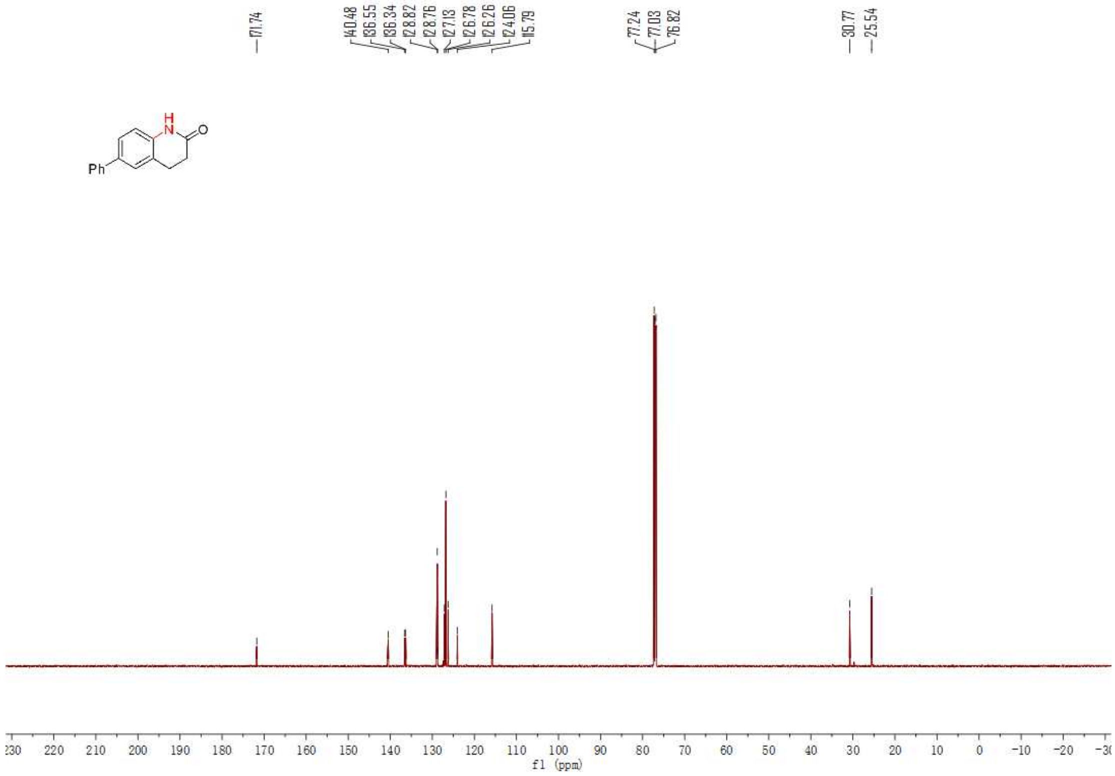

^{1}H NMR spectrum of compound 2e (600 MHz, CDCl3)

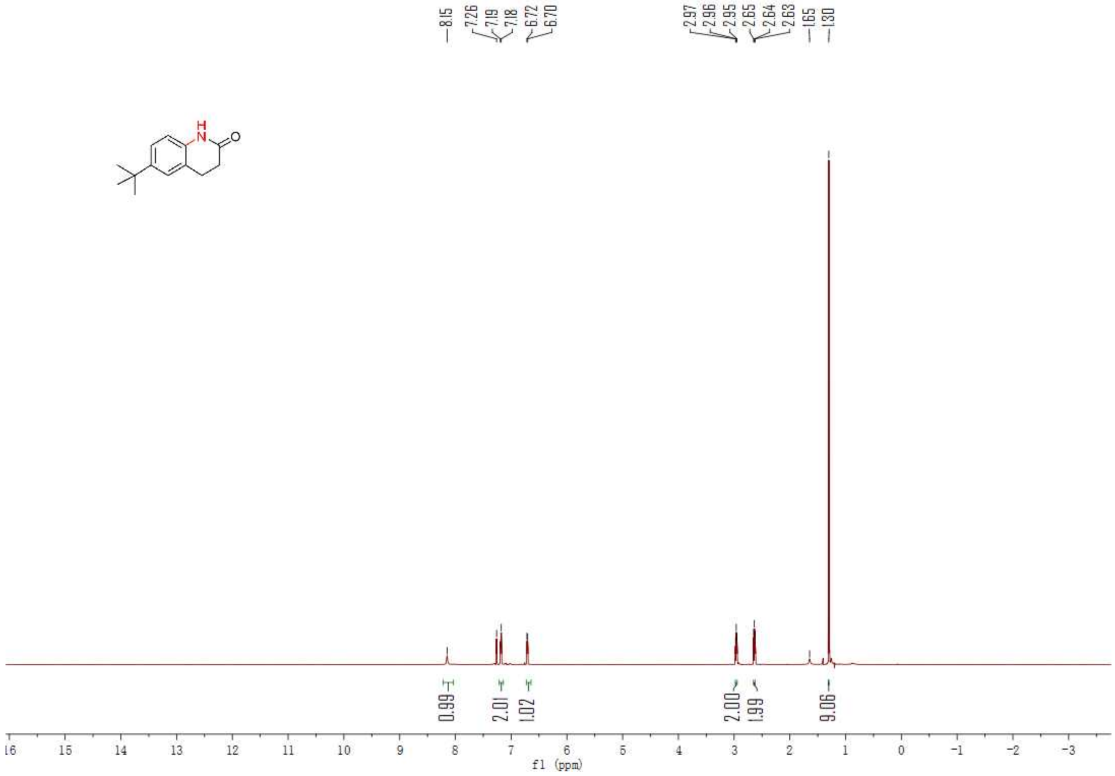

^{13}C{^{1}H} NMR spectrum of compound 2e (150 MHz, CDCl3)

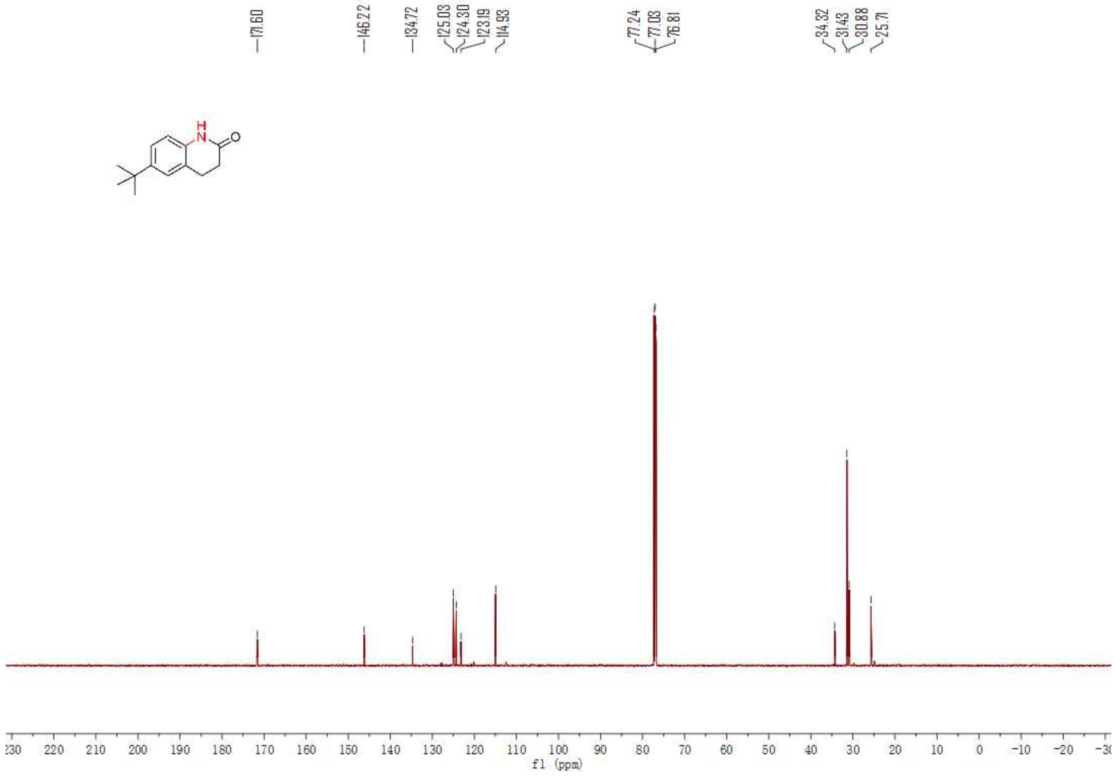

^{1}H NMR spectrum of compound 2f (600 MHz, $CDCl_3$)

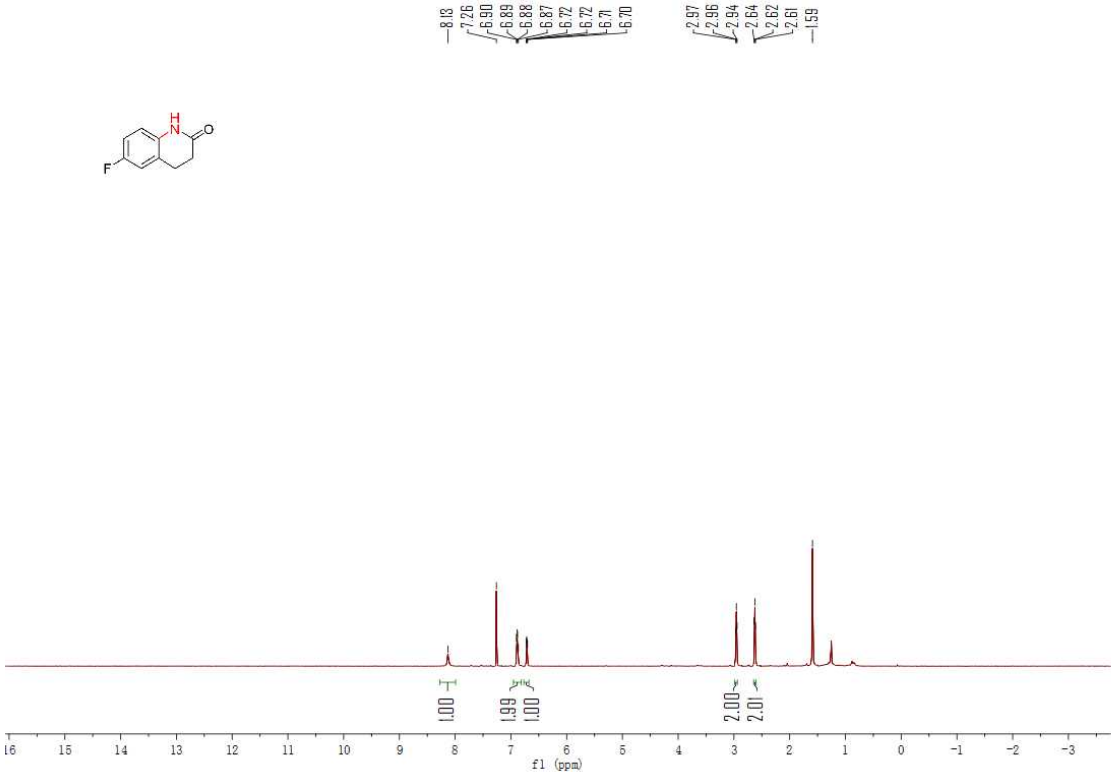

$^{13}C\{^{1}H\}$ NMR spectrum of compound 2f (150 MHz, $CDCl_3$)

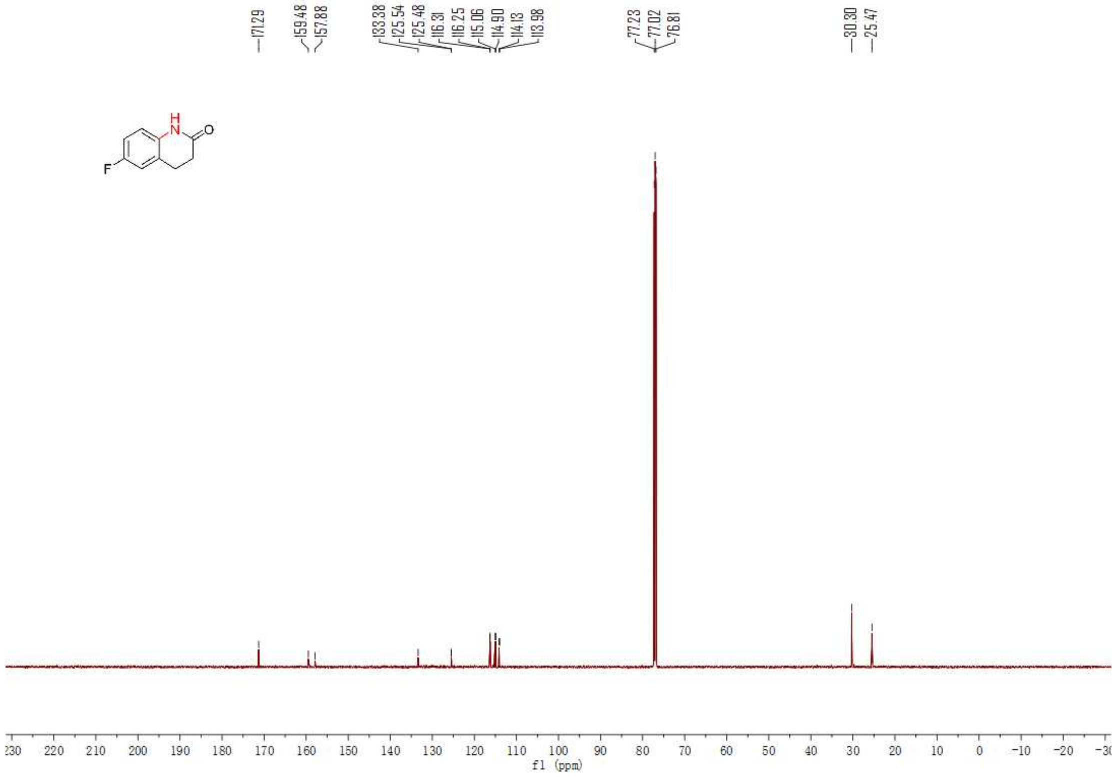

^{19}F NMR spectrum of compound 2f (565 MHz, $CDCl_3$)

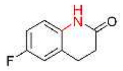

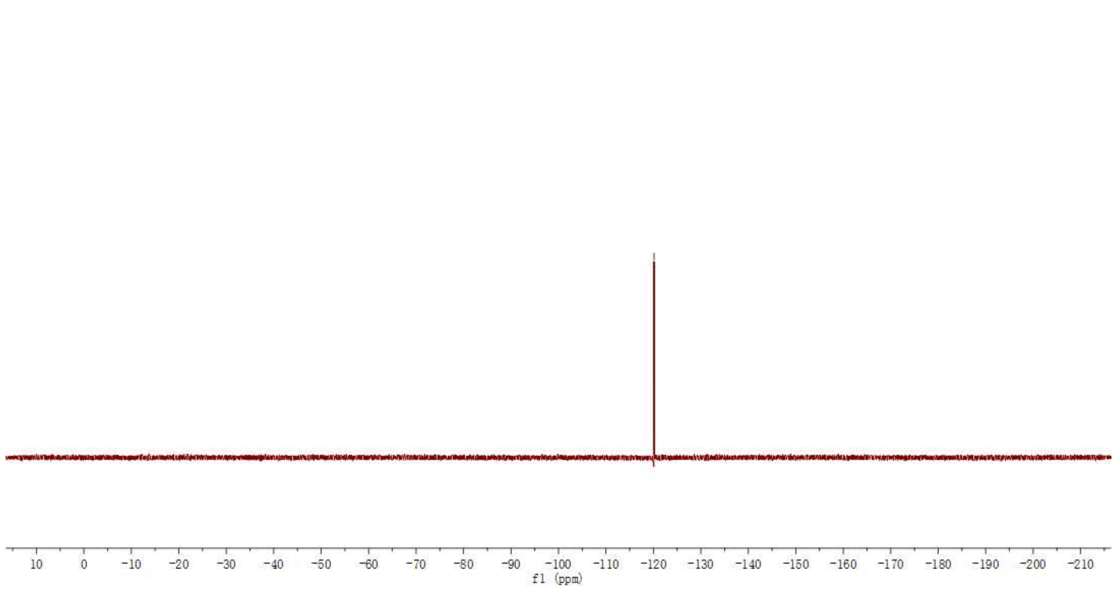

^{1}H NMR spectrum of compound 2g (600 MHz, $CDCl_3$)

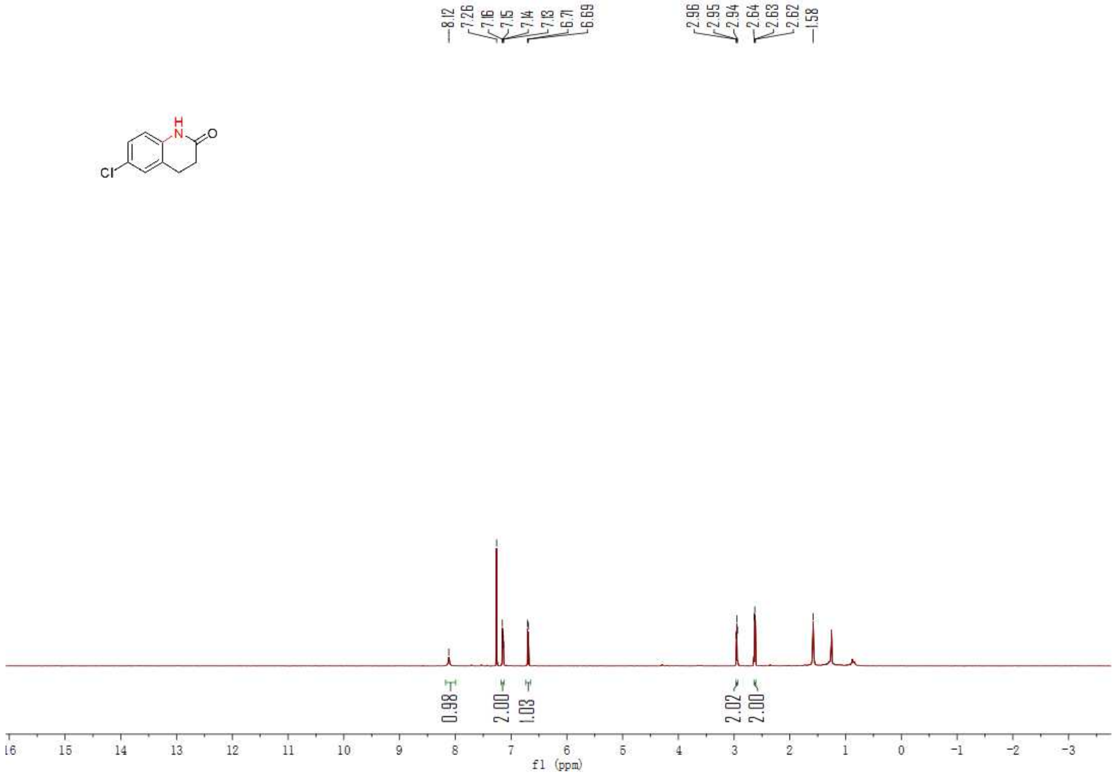

$^{13}C\{^{1}H\}$ NMR spectrum of compound 2g (150 MHz, $CDCl_3$)

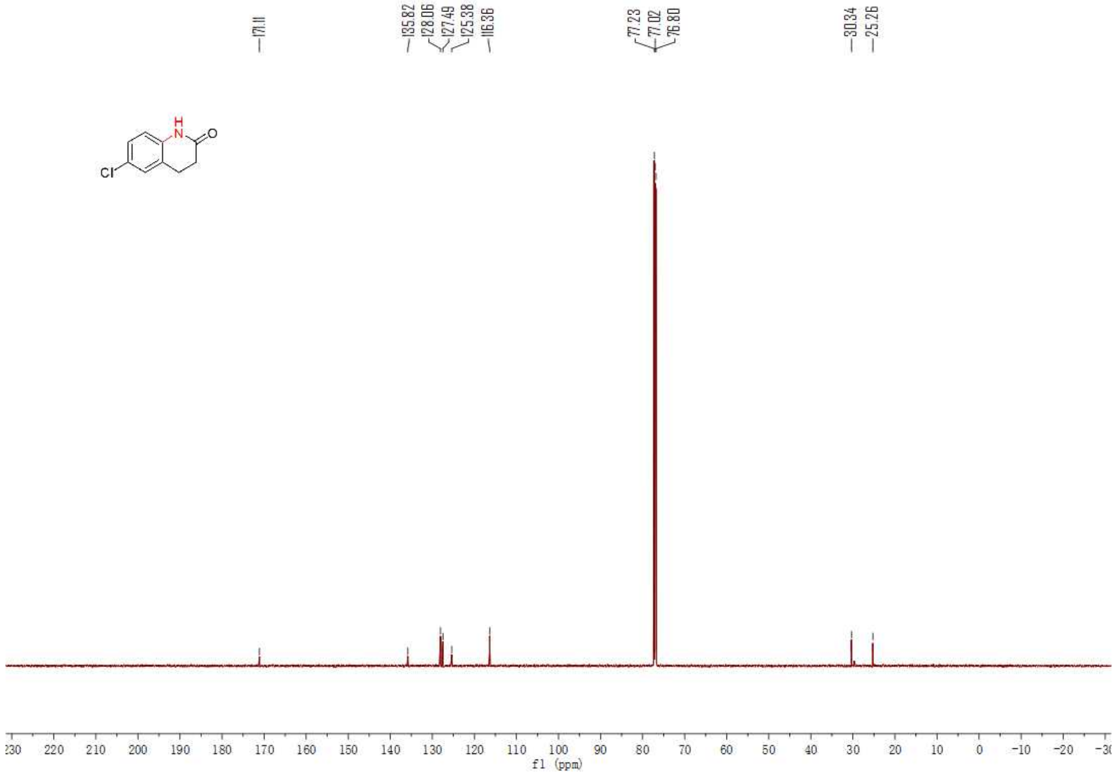

^{1}H NMR spectrum of compound 2h (600 MHz, $CDCl_3$)

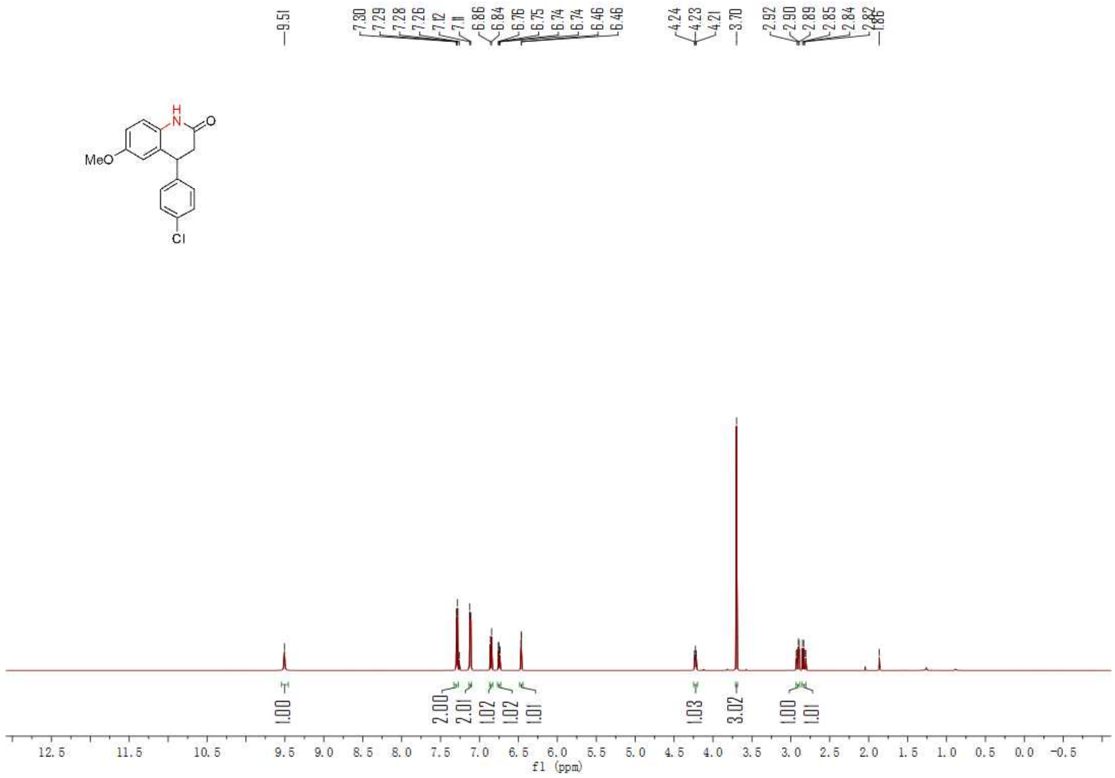

$^{13}C\{^{1}H\}$ NMR spectrum of compound 2h (150 MHz, $CDCl_3$)

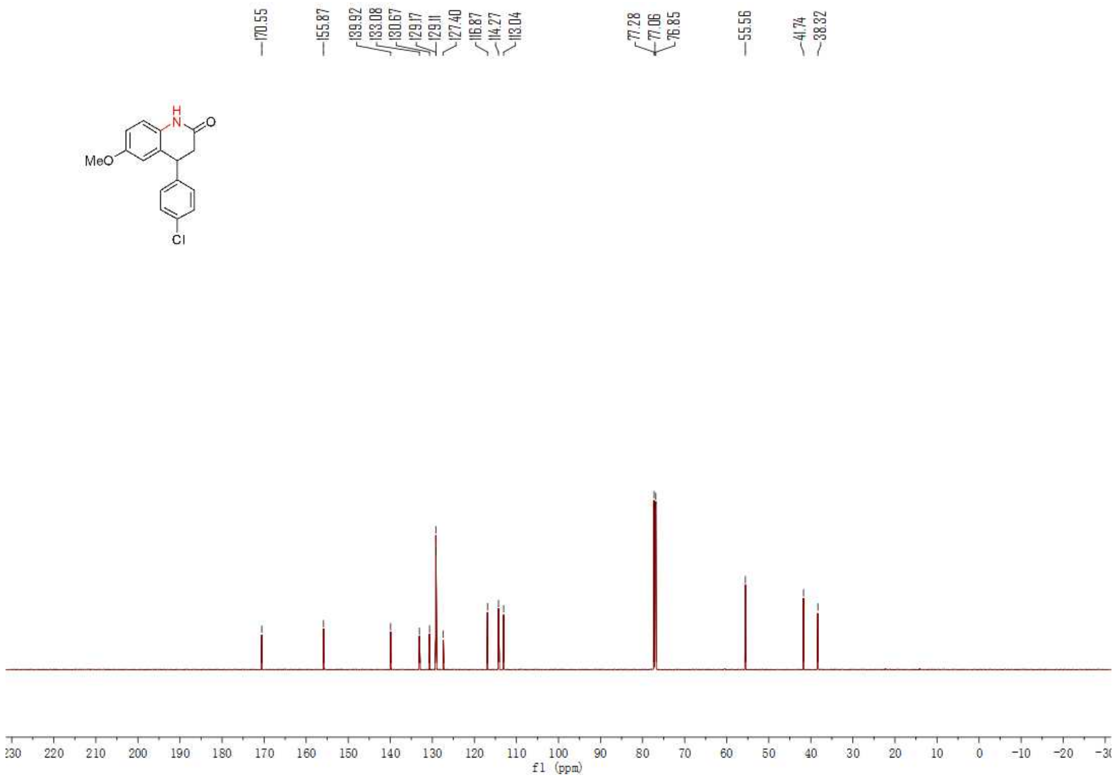

^{1}H NMR spectrum of compound 2i (600 MHz, $CDCl_3$)

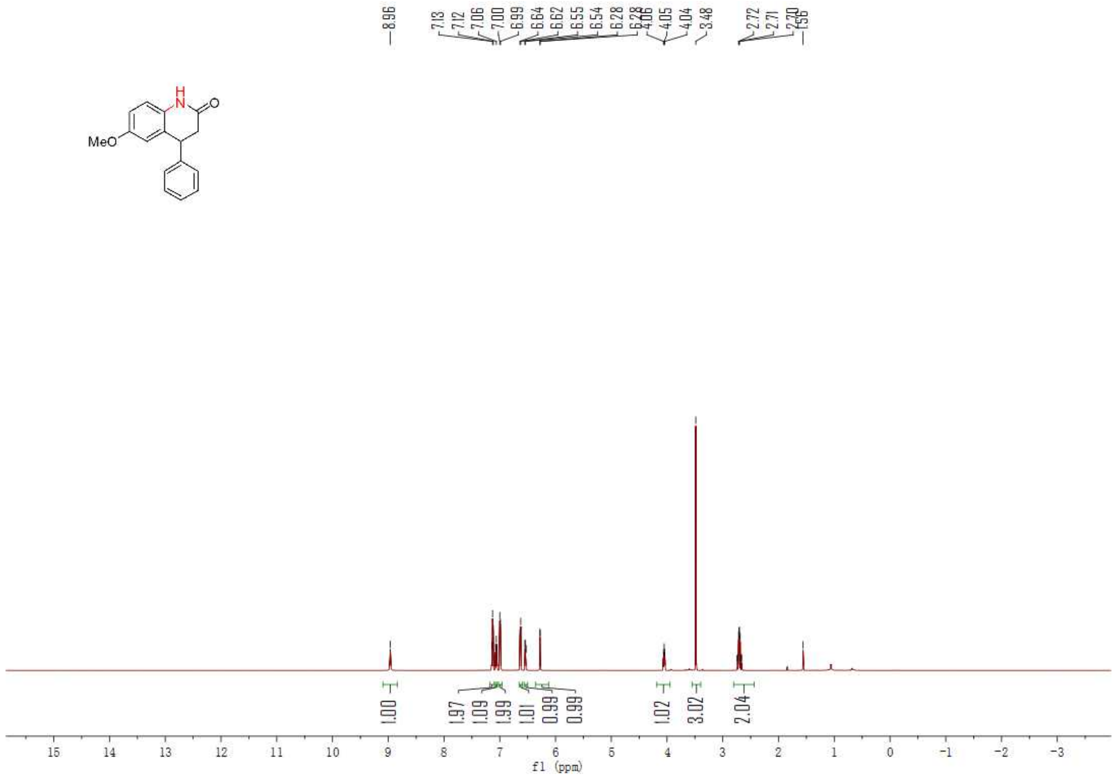

$^{13}C\{^{1}H\}$ NMR spectrum of compound 2i (150 MHz, $CDCl_3$)

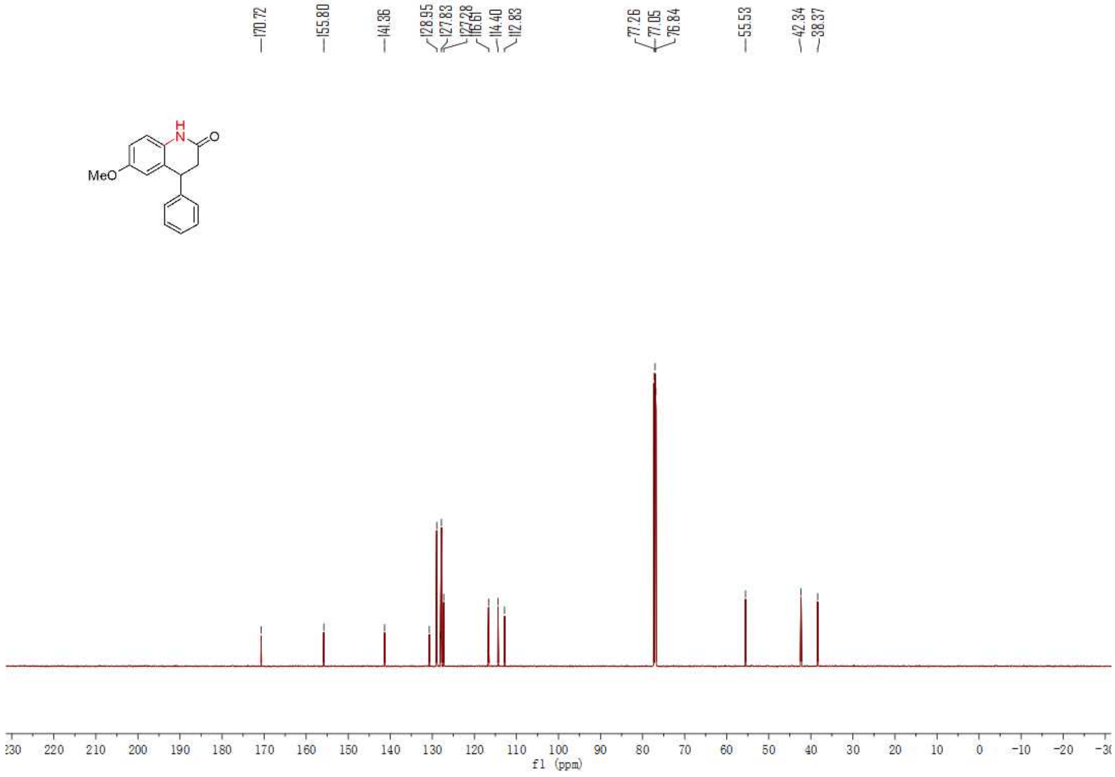

^{1}H NMR spectrum of compound 2k (600 MHz, $CDCl_3$)

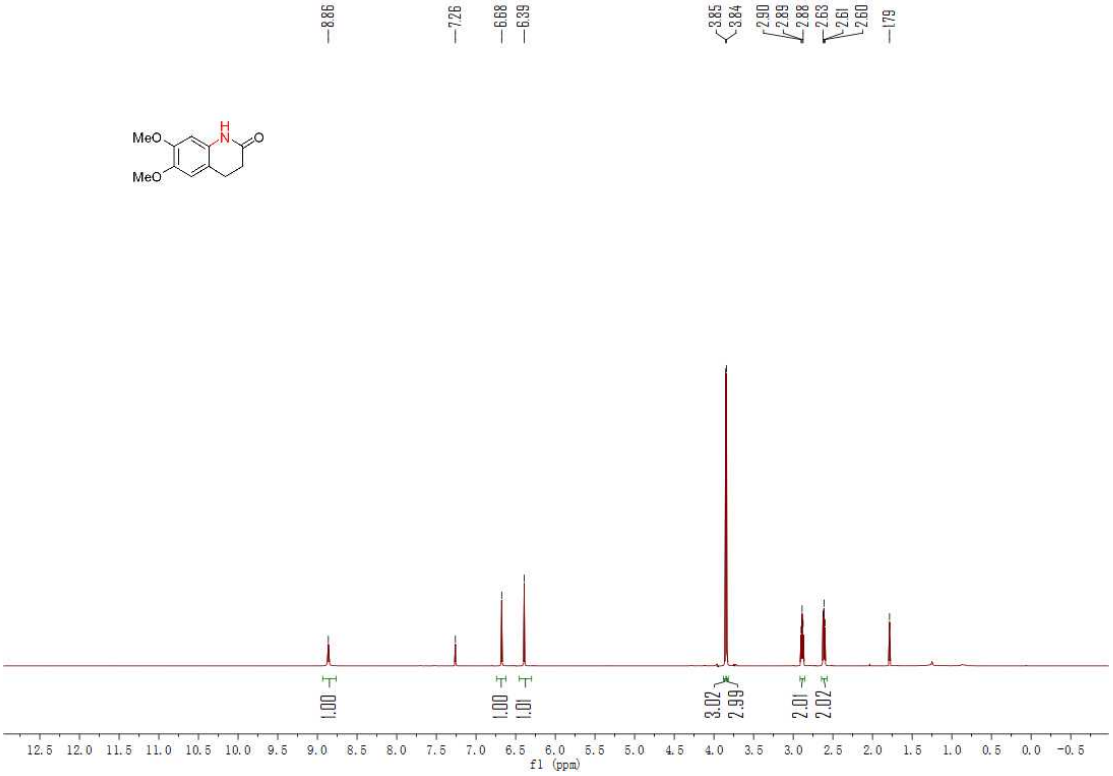

^{13}C{^{1}H} NMR spectrum of compound 2k (150 MHz, $CDCl_3$)

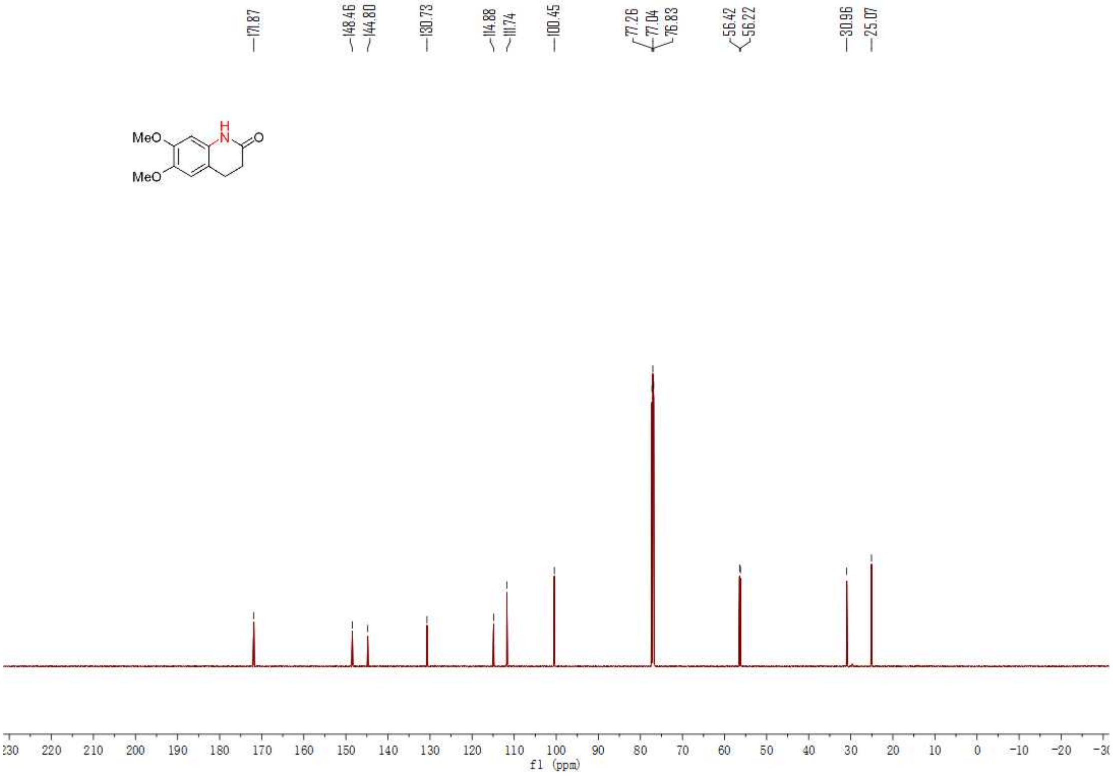

^{1}H NMR spectrum of compound 2l (600 MHz, $CDCl_3$)

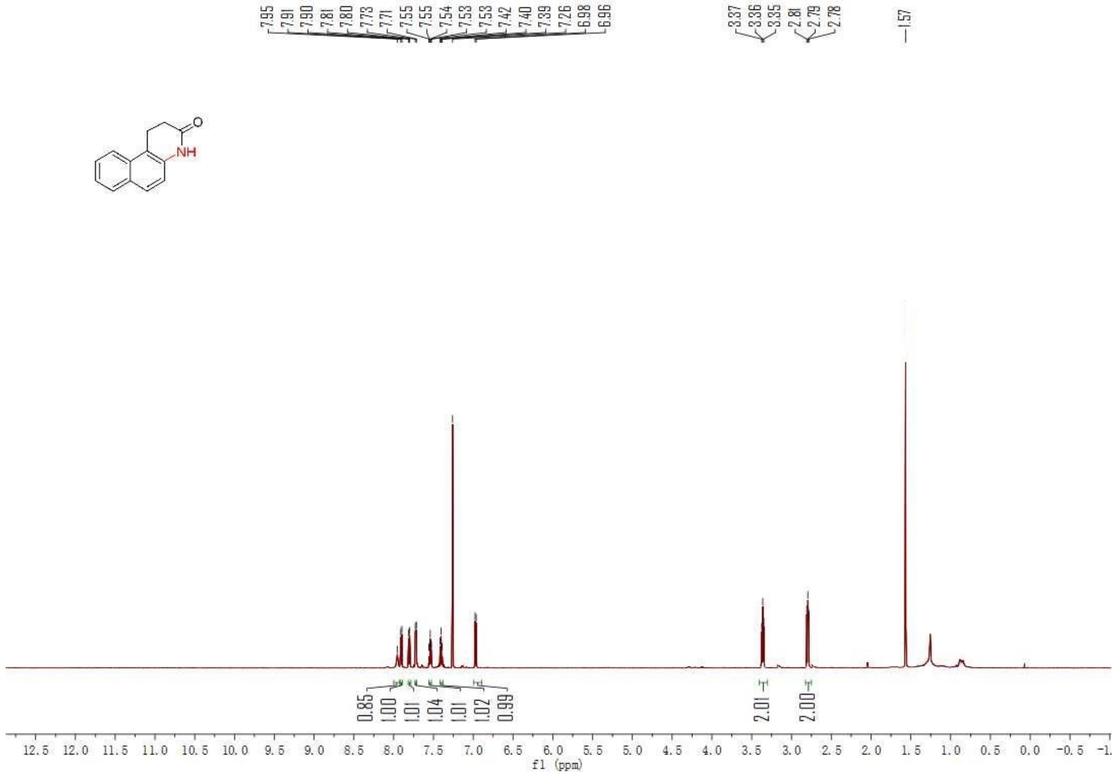

$^{13}C\{^{1}H\}$ NMR spectrum of compound 2l (150 MHz, $CDCl_3$)

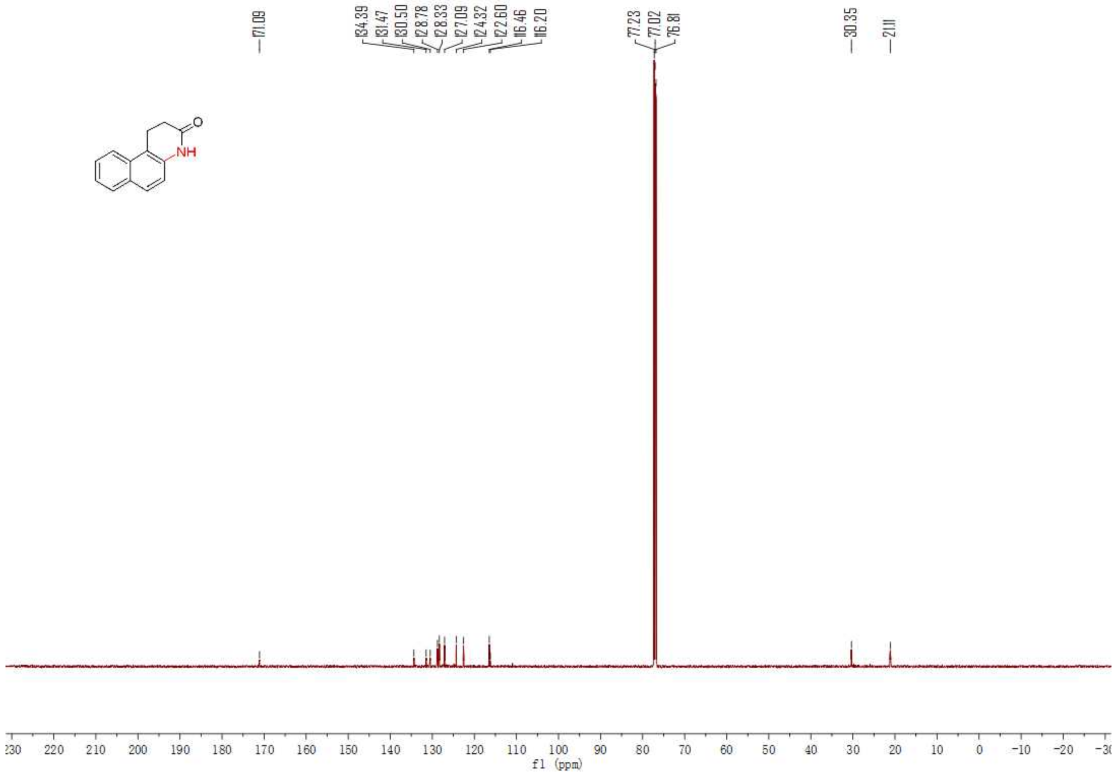

^{1}H NMR spectrum of compound 2l'a and 2l'b (600 MHz, CDCl$_3$)

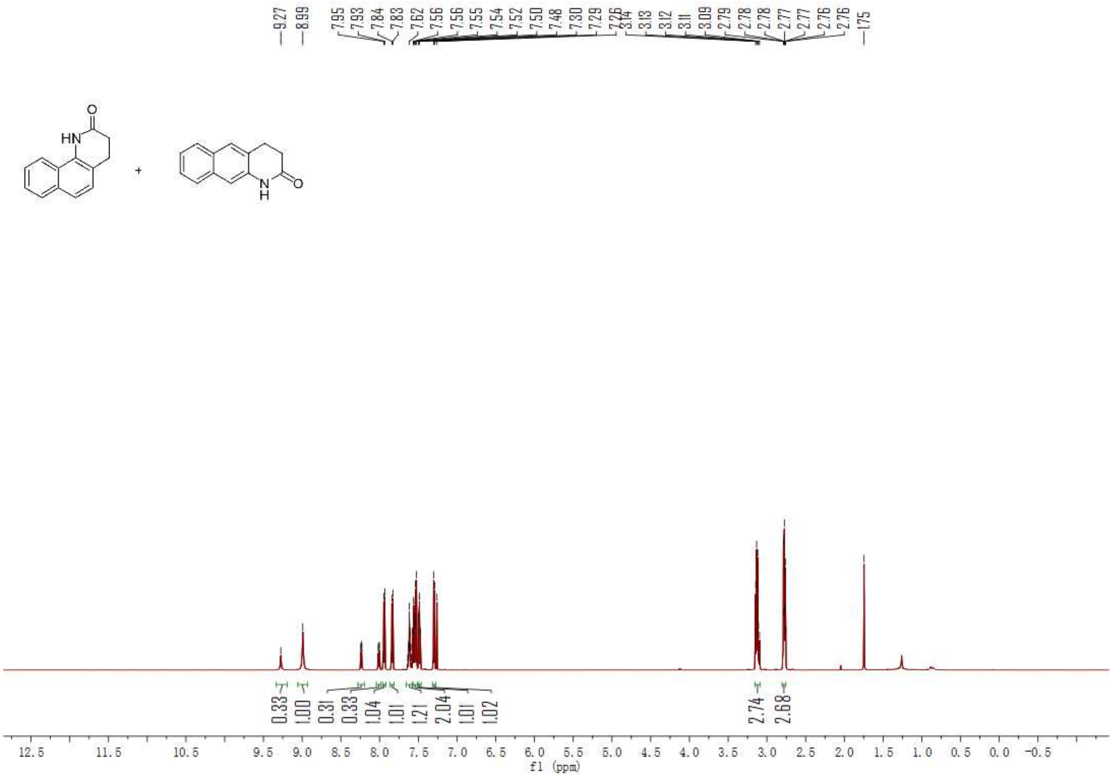

^{13}C{^{1}H} NMR spectrum of compound 2l'a and 2l'b (150 MHz, CDCl$_3$)

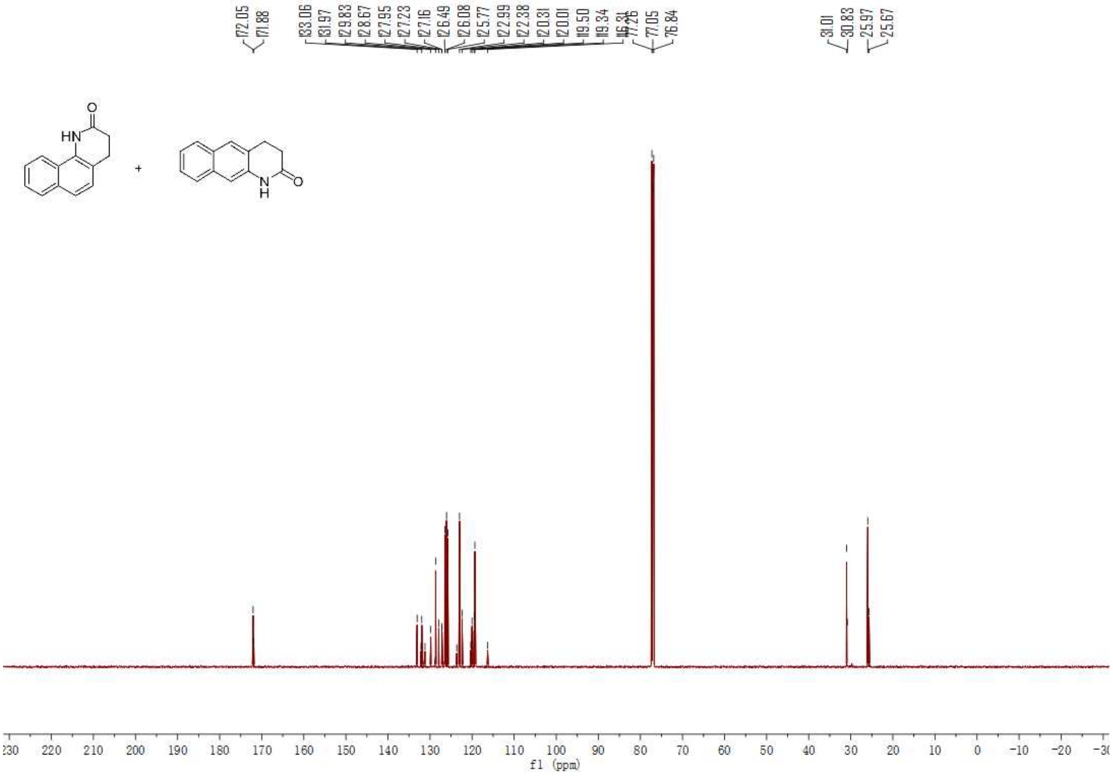

^{1}H NMR spectrum of compound 2m and 2m' (600 MHz, $CDCl_3$)

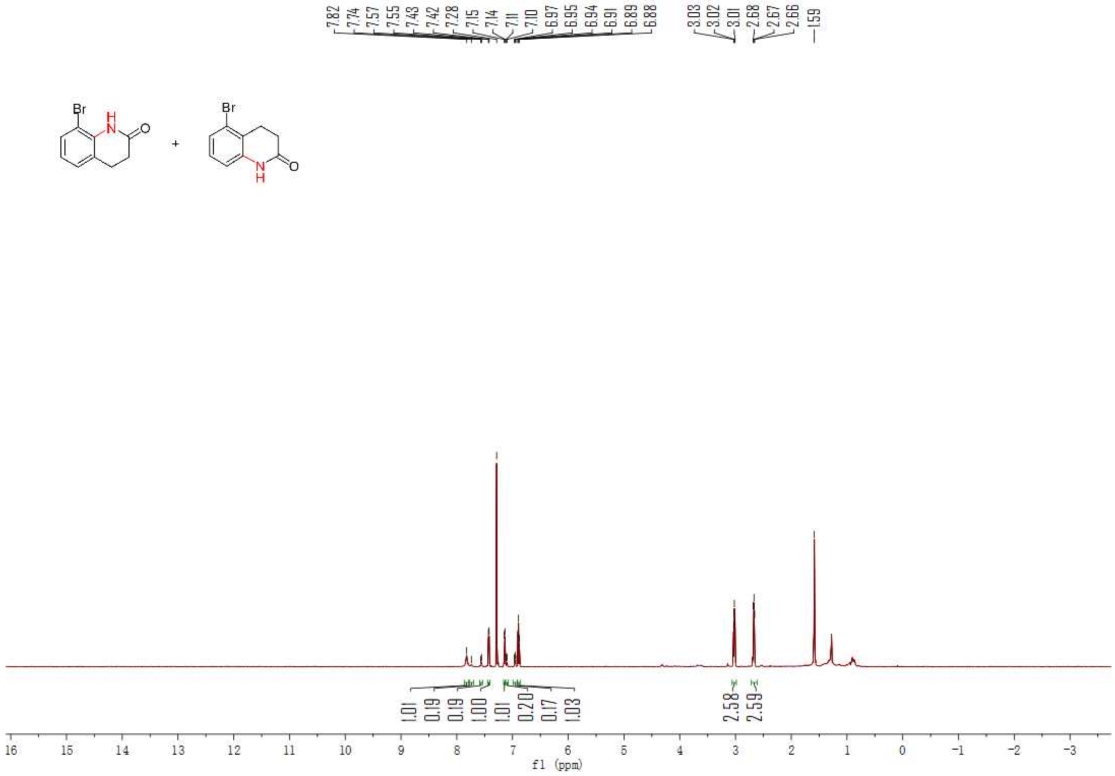

^{13}C{^{1}H} NMR spectrum of compound 2m and 2m' (150 MHz, $CDCl_3$)

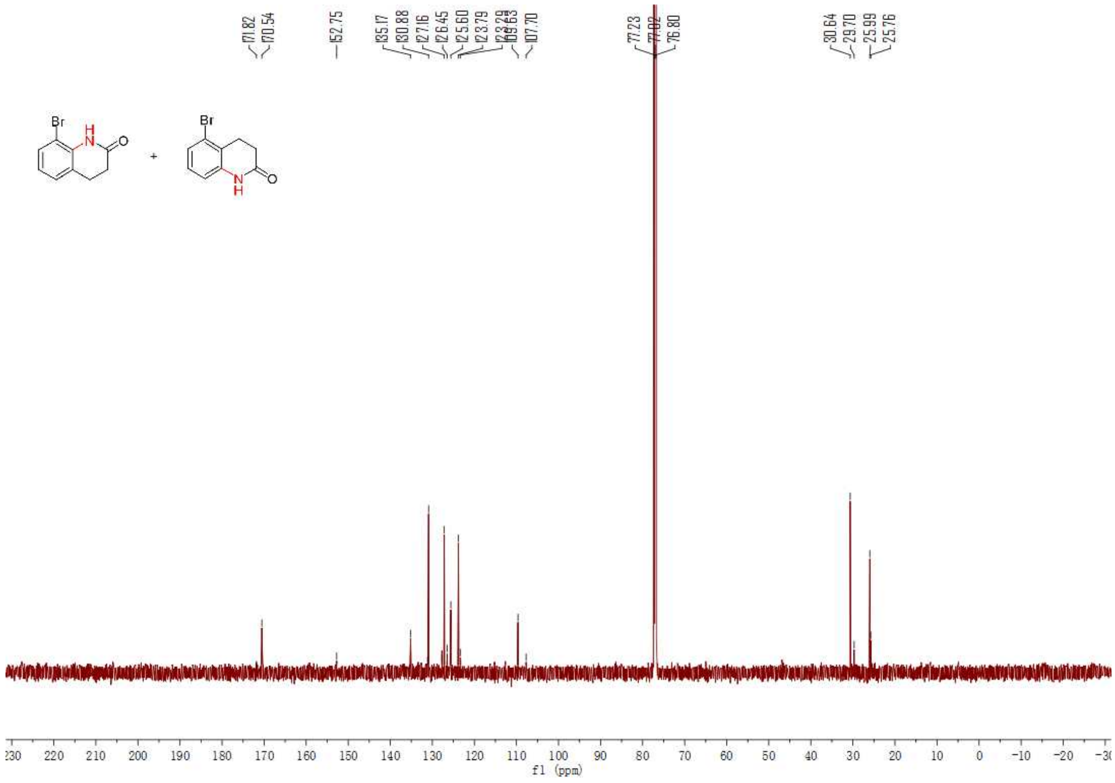

^{1}H NMR spectrum of compound 2n (400 MHz, $CDCl_3$)

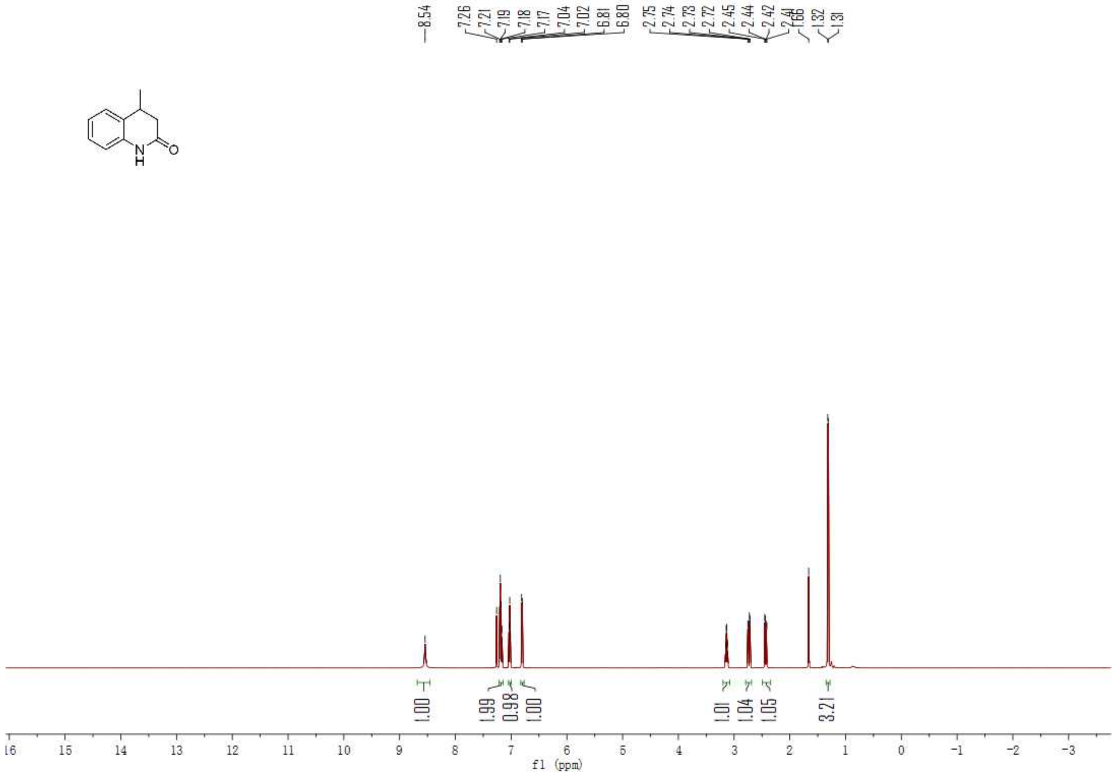

^{13}C{^{1}H} NMR spectrum of compound 2n (101 MHz, $CDCl_3$)

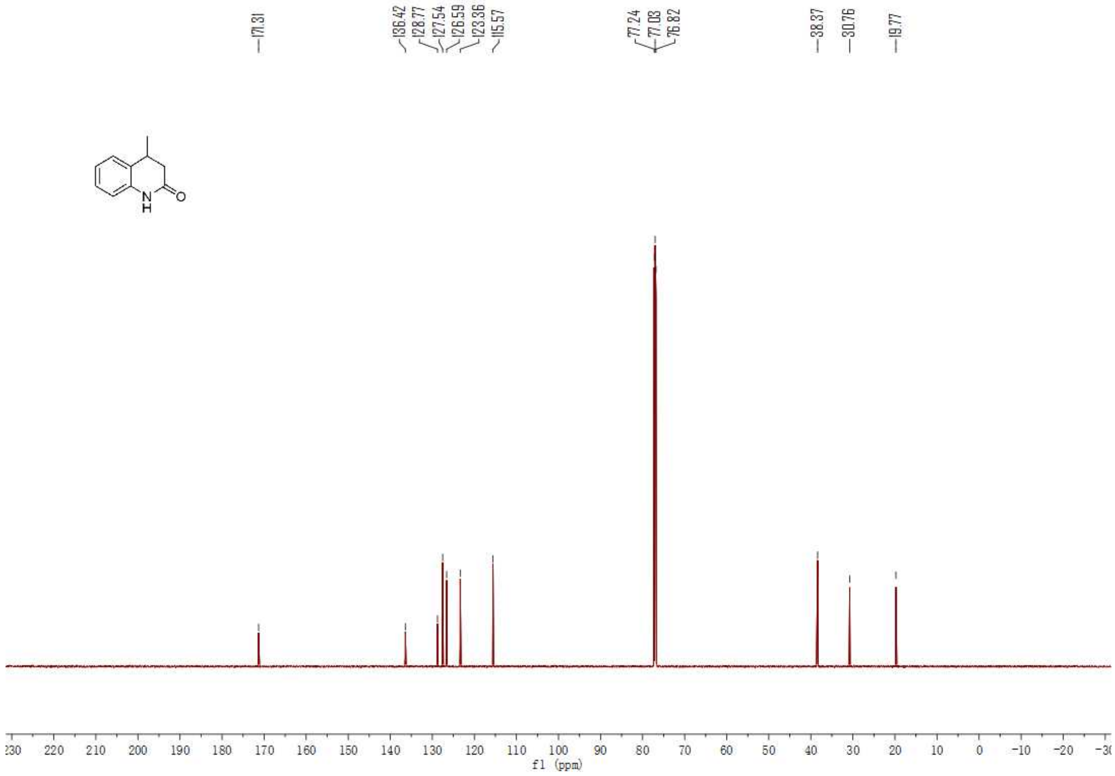

¹H NMR spectrum of compound 2o (600 MHz, $CDCl_3$)

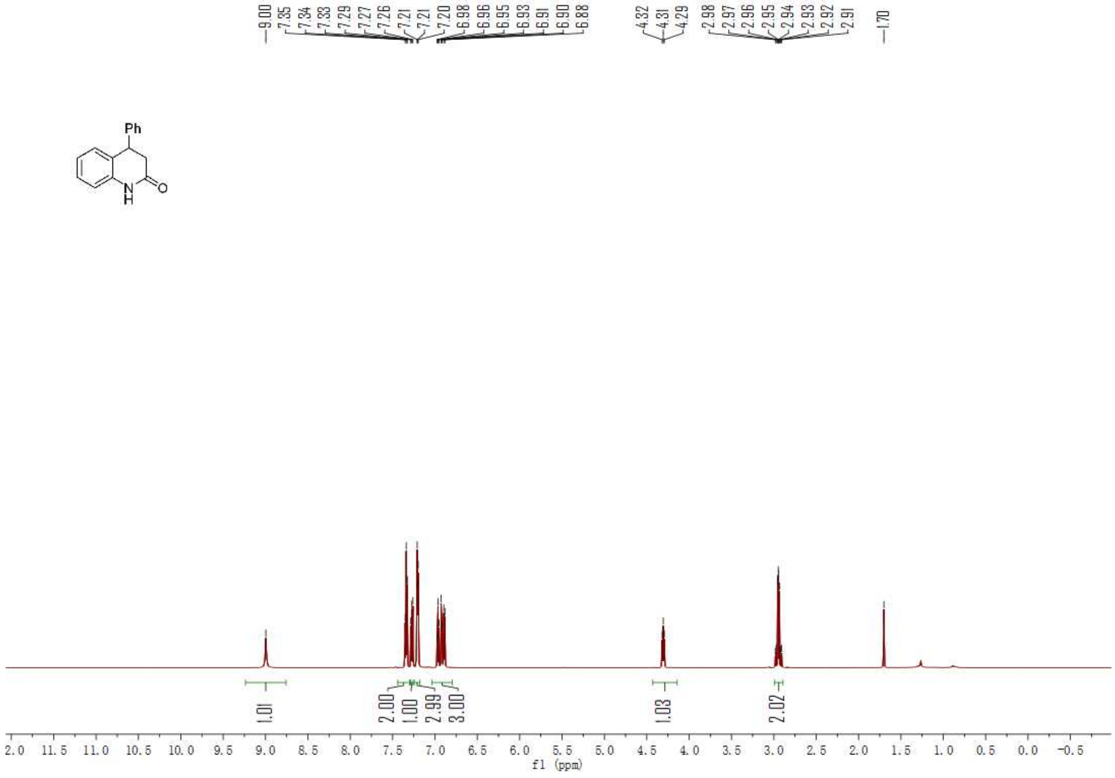

¹³C{¹H} NMR spectrum of compound 2o (150 MHz, $CDCl_3$)

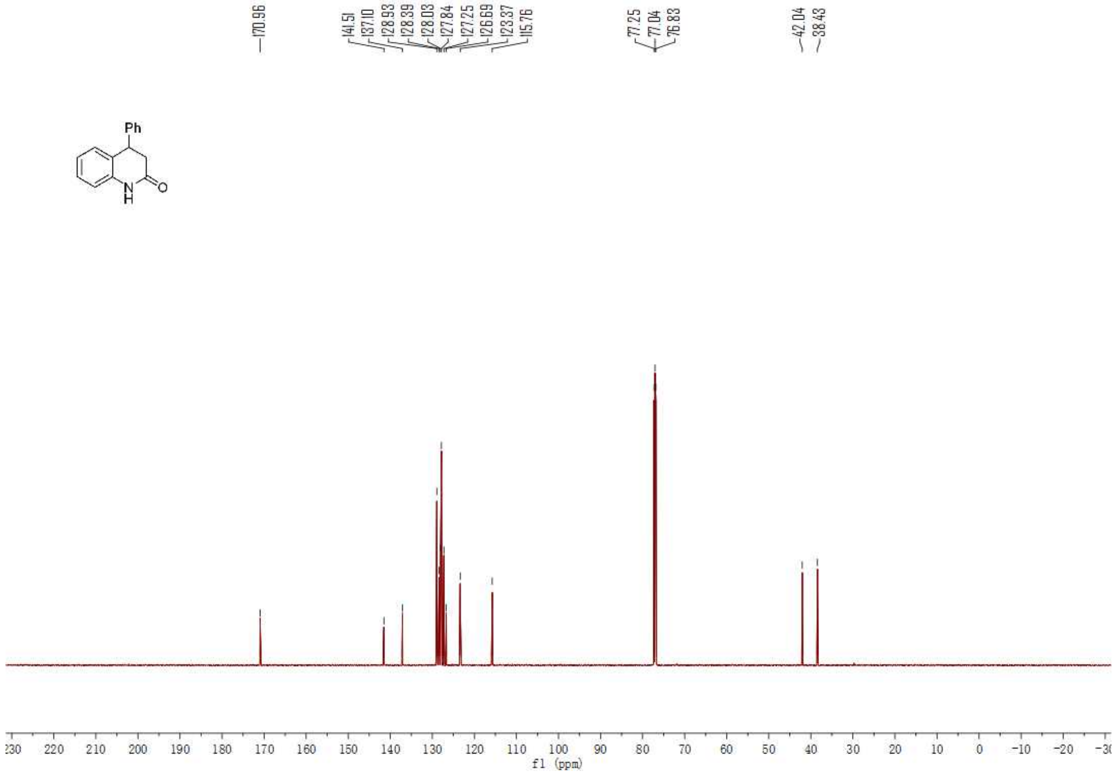

^{1}H NMR spectrum of compound 3 (600 MHz, $CDCl_3$)

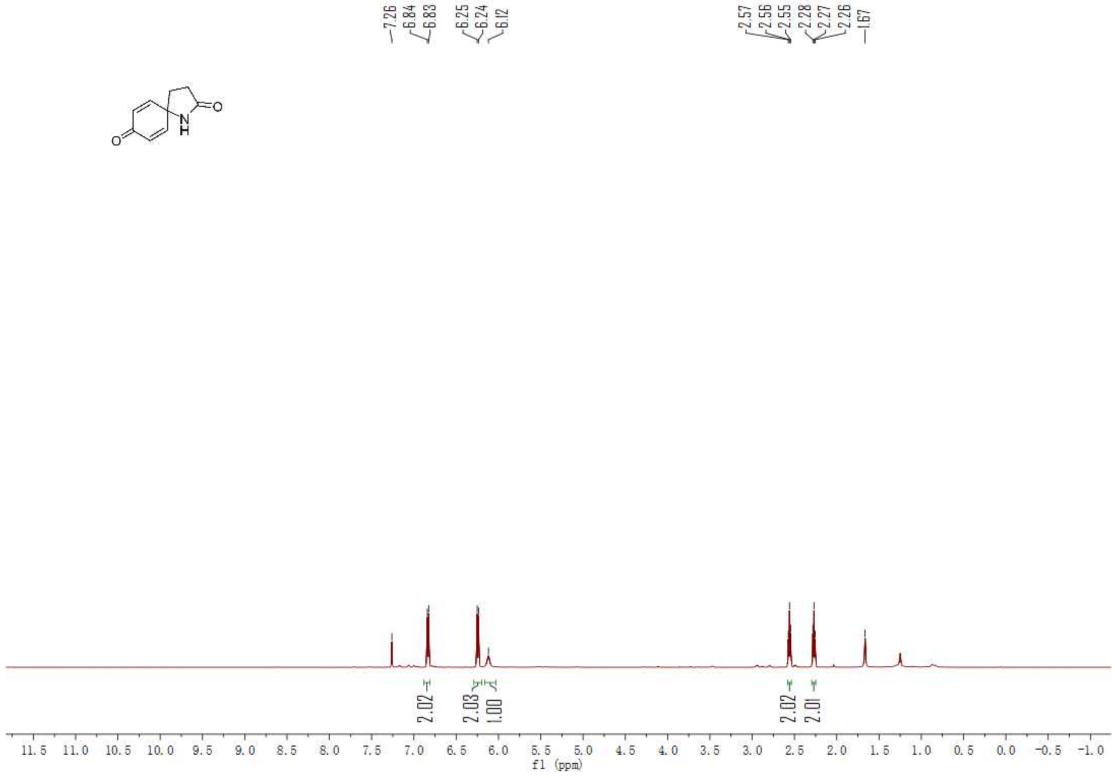

^{13}C{^{1}H} NMR spectrum of compound 3 (150 MHz, $CDCl_3$)

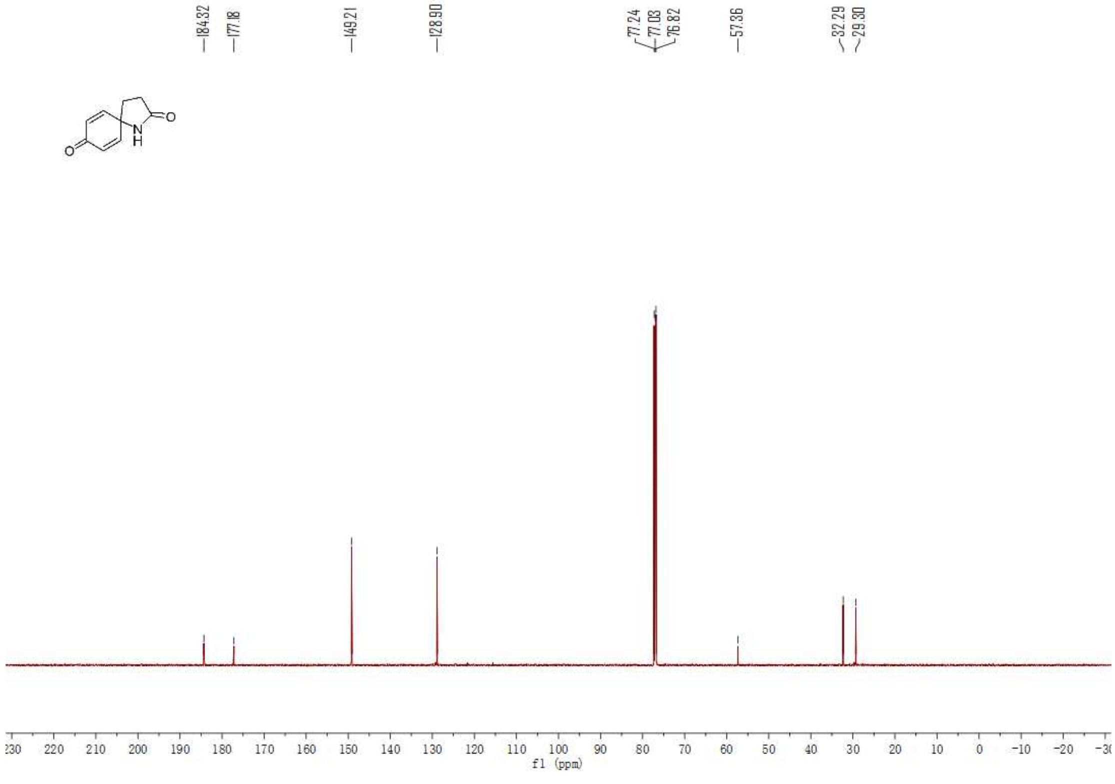

Supplement: Supplementary file 1 — Supporting Information [file ANIE-61-0-s001.pdf]
